# Heteromultimeric sarbecovirus receptor binding domain immunogens primarily generate variant-specific neutralizing antibodies

**DOI:** 10.1073/pnas.2317367120

**Published:** 2023-12-14

**Authors:** Trinity Zang, Edmund Osei Kuffour, Viren A. Baharani, Marie Canis, Fabian Schmidt, Justin Da Silva, Alexander Lercher, Pooja Chaudhary, Hans-Heinrich Hoffmann, Anna Gazumyan, Ileana C. Miranda, Margaret R. MacDonald, Charles M. Rice, Michel C. Nussenzweig, Theodora Hatziioannou, Paul D. Bieniasz

**Affiliations:** ^a^Laboratory of Retrovirology, The Rockefeller University, New York, NY 10065; ^b^HHMI, The Rockefeller University, New York, NY 10065; ^c^Laboratory of Molecular Immunology, The Rockefeller University, New York, NY 10065; ^d^Laboratory of Virology and Infectious Diseases, The Rockefeller University, New York, NY 10065; ^e^Laboratory of Comparative Pathology, The Rockefeller University, New York, NY 10065

**Keywords:** vaccine, vaccinology, antibodies, SARS-CoV-2

## Abstract

Vaccination will likely be a key component of strategies to curtail or prevent future sarbecovirus pandemics and to reduce the prevalence of infection and disease by future SARS-CoV-2 variants. A “pan-sarbecovirus” vaccine, that provides maximum possible mitigation of human disease, should elicit neutralizing antibodies with maximum possible breadth. By positioning multiple different receptor binding domain (RBD) antigens in close proximity on a single immunogen, it is postulated that cross-reactive B cell receptors might be selectively engaged. Heteromultimeric vaccines could therefore elicit individual antibodies that neutralize a broad range of viral species. Here, we use model systems to investigate the ability of multimeric sarbecovirus RBD immunogens to expand cross-reactive B cells and elicit broadly reactive antibodies. Homomultimeric RBD immunogens generated higher serum neutralizing antibody titers than the equivalent monomeric immunogens, while heteromultimeric RBD immunogens generated neutralizing antibodies recognizing each RBD component. Moreover, RBD heterodimers elicited a greater fraction of cross-reactive germinal center B cells and cross-reactive RBD binding antibodies than did homodimers. However, when serum antibodies from RBD heterodimer-immunized mice were depleted using one RBD component, neutralization activity against the homologous viral pseudotype was removed, but neutralization activity against pseudotypes corresponding to the other RBD component was unaffected. Overall, simply combining divergent RBDs in a single immunogen generates largely separate sets of individual RBD-specific neutralizing serum antibodies that are mostly incapable of neutralizing viruses that diverge from the immunogen components.

## Significance

Multimeric vaccines containing variant proteins from related viruses are expected to generate broader neutralizing antibody responses. Combining variants in a single heteromultimeric immunogen particle or polypeptide may preferentially stimulate B cells that recognize multiple viral variants and subsequently produce cross-reactive antibodies. We show that cross-reactive B cells and antibodies can indeed be elicited using immunogens built from two distinct SARS-like coronaviruses receptor binding domains. However, most antibodies with virus neutralizing activity generated by such immunogens recognize viruses corresponding to only one of the two immunogen components. This is likely because sequence variation is concentrated in areas recognized by neutralizing antibodies; thus, the cross-reactive B cells mostly recognize sequences conserved in multiple viral variants and primarily generate antibodies lacking neutralizing activity.

Coronaviruses of the sarbecovirus subgenus, such as SARS-CoV and SARS-CoV-2, have demonstrated a capacity to generate epidemics and pandemics following spillover into humans (1). Shortly after the development of SARS-CoV-2 vaccines, when immunogens and ancestral circulating viral strains were near-perfectly matched, monovalent mRNA vaccines could effectively prevent SARS-CoV-2 transmission (2, 3), offering the prospect of vaccine-induced “herd immunity.” However, subsequent waves of infection and SARS-CoV-2 antigenic drift have led to viral escape from neutralizing antibodies elicited by earlier variants (4–8). Consequently, the ability of ancestral variant-based vaccines to prevent transmission has been eroded, although they retain substantial effectiveness in preventing severe disease (9, 10).

Vaccination will likely be a key component of strategies to curtail or prevent future sarbecovirus pandemics and to reduce the prevalence of infection and disease by future SARS-CoV-2 variants. A “pan-sarbecovirus” vaccine, that provides maximum possible mitigation of human disease, should elicit antibodies with maximum possible breadth, ideally with pan-sarbecovirus neutralizing activity (11). Such a vaccine might prevent transmission of new SARS-CoV-2 variants and curtail nascent epidemics of sarbecoviruses that may emerge from animal reservoirs. However, antigenic variation among animal sarbecoviruses is considerable, and antigenic drift in SARS-CoV-2 is ongoing (12).

Neutralizing antibody-based protection against diverse sarbecoviruses could, in principle, be achieved by vaccination in distinct ways. In one scenario, many different, narrowly specific antibodies would provide protection against many well-matched extant viruses (11). This scenario would necessitate the administration of many, or highly multivalent, vaccines. While potentially effective against known virus threats, such a strategy would be unlikely to provide protection against any divergent virus or variant whose emergence was not anticipated and thus was not included in the vaccine cocktail. In another scenario, protection could be provided by a smaller number of broadly neutralizing antibody lineages that are effective against many different viruses with diverse sequences (13–16). Such antibodies would need to target invariant neutralizing epitopes or tolerate sequence variation in the targeted epitopes. Broadly cross-reactive antibodies would be more likely to be capable of neutralizing viruses whose spillover was unanticipated. However, such a scenario would require the development of vaccines that are capable of efficiently generating such broadly cross-reactive neutralizing antibodies.

In the case of sarbecoviruses, the spike protein receptor binding domain (RBD) constitutes the major target of potent neutralizing antibodies and includes epitopes that vary to differing degrees among sarbecoviruses (5, 17). For example, the majority of antibodies elicited by SARS-CoV-2 do not neutralize SARS-CoV, but a subset that neutralizes both SARS-CoV and SARS-CoV-2 target less variable portions of the RBD (15, 18, 19). Notably, SARS-CoV-2 RBD-specific neutralizing antibodies in the B cell memory compartment can acquire breadth during months of affinity maturation following infection and vaccination (20–23) and, thus, the ability to neutralize SARS-CoV-2 variants or other sarbecoviruses (such as SARS-CoV) whose epitope target sequences differ from the original antigen (24). Similarly, after multiple antigen exposures, polyclonal sera from SARS-CoV or SARS-CoV-2 infected and later vaccinated individuals can neutralize SARS-CoV, other sarbecoviruses and some SARS-CoV-2 variants (17, 25, 26).

Heteromultimeric vaccines such as mosaic nanoparticles could potentially selectively elicit individual antibodies with the ability to neutralize a broad range of viral species by positioning multiple different antigens in close proximity on a single immunogen (27–32). In principle, selective stimulation and expansion of B cells whose antigen receptors target conserved epitopes or, following diversification, are preferentially cross-linked by heteromultimeric antigens might be achieved. Here, we use model systems to investigate the ability of multimeric sarbecovirus RBD immunogens to elicit cross-reactive B cells and generate broadly cross-reactive serum antibodies.

## Results

### Heteromultimeric Sarbecovirus RBD Immunogens.

**Fig. 1.**
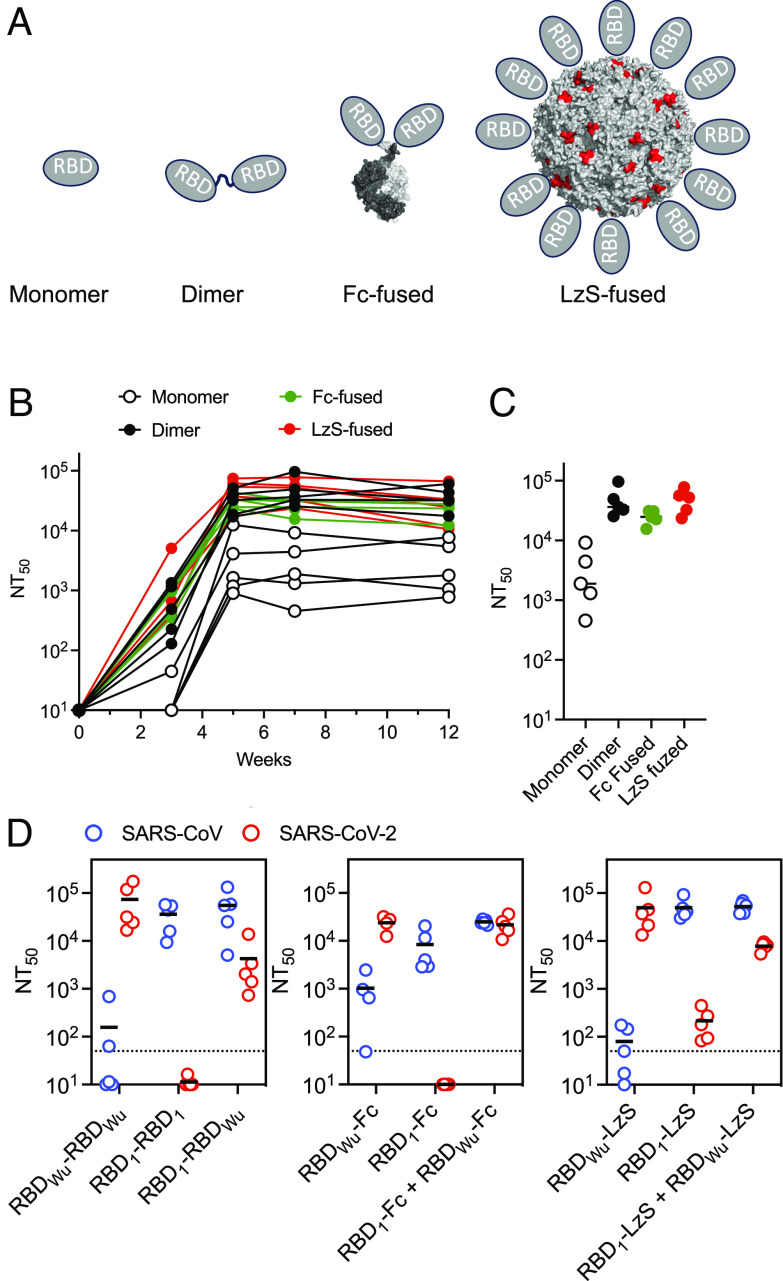
Multimeric sarbecovirus RBD immunogens. (*A*) Schematic representation of genetically fused RBD dimers, RBD-IgG Fc fusions, and LzS nanoparticles. For the LzS nanoparticles, the N terminus of each LzS monomer RBD fusion site is indicated in red. (*B*) SARS-CoV-2_Wu_ pseudotype neutralizing titers in mouse sera following a 2-dose immunization with the indicated proteins (week zero = first dose, week 3 = second dose), n = 5 individual mice for each immunogen. (*C*) SARS-CoV-2_Wu_ pseudotype neutralizing titers in mouse sera (pooled week 7/8 bleeds) following a 2-dose immunization with the indicated proteins. (*D*) SARS-CoV and SARS-CoV-2_Wu_ pseudotype neutralizing titers in mouse sera at week 11/12 following initiation of a 2-dose immunization with the indicated homomultimeric or heteromultimeric RBD dimers (*Left*), RBD-IgG fusions (*Center*) and LzS nanoparticles (*Right*). In (*C* and *D*), horizontal lines indicate group means, and symbols indicate individual mice n = 5 per group. The dotted line indicates the lowest sera dilution tested (1:50).

We first compared the ability of monomeric and multimeric homotypic SARS-CoV-2 RBD-based immunogens to elicit neutralizing antibodies. A SARS-CoV-2 RBD protein from the Wuhan-hu-1 strain (RBD_Wu_) was expressed as a monomeric protein, as a genetically fused single polypeptide dimer (RBD_Wu_-RBD_Wu_), or a dimer by fusion to an IgG1 Fc domain, or as a high order 60-mer nanoparticle by fusion to a modified Lumazine Synthase (LzS) (33) (Fig. 1*A* and *SI Appendix*, Fig. S1*A*). Mice were inoculated with each protein immunogen at week 0 and week 3. Neutralizing titers against SARS-CoV-2_Wu_ pseudotypes plateaued at ~week 5 and remained constant until at least week 12 (Fig. 1*B*). Even though the same total mass of protein was given in all cases, each of multimeric proteins generated mean SARS-CoV-2 pseudotype neutralization titers that were sevenfold to 14-fold greater than those generated by the monomeric RBD_Wu_ protein (*P* = 0.0079, Fig. 1*C*). The homomultimeric RBD_Wu_ immunogens generated similar neutralizing antibody titers, irrespective of how multimerization was mediated.

We next generated a fusion dimer, Fc and LzS based homomultimeric immunogens containing the RBD of SARS-CoV (RBD_1_) as well as heteromultimeric immunogens containing both RBD_1_ and RBD_Wu_ (*SI Appendix*, Fig. S1 *A* and *B*) and compared the ability of homomultimers and heteromultimers to generate antibodies that neutralized SARS-CoV and SARS-CoV-2_Wu_. The mean NT_50_ values elicited by RBD_1_ homomultimers were 8346 to 49464 against SARS-CoV but only <10 to 215 against SARS-CoV-2_Wu_ (Fig. 1*D*). Conversely, NT_50_ values generated by RBD_Wu_ homomultimers were 23,870 to 73,325 against SARS-CoV-2_Wu_ but only 79 to 1,027 against SARS-CoV (Fig. 1*D*). The heteromultimers combining both RBD_1_ and RBD_Wu_, elicited high titer neutralizing antibodies against both viruses (mean NT_50_ = 24,840 to 55,079 against SARS-CoV and 4,257 to 21,675 against SARS-CoV-2_Wu_) (Fig. 1*D*). Overall, the homomultimeric immunogens elicited neutralizing antibodies that were largely specific for the autologous virus, while the heteromultimers elicited antibodies that neutralized both viruses.

### Expansion of Cross-Reactive B Cells by RBD Heterodimers.

Although each of the various heteromultimeric immunogens generated similar neutralization titers against both SARS-CoV and SARS-CoV-2_Wu_ (Fig. 1*D*), the Fc fused and LzS nanoparticle fused RBD proteins differ from the fusion dimer in that they generate a mixture of heteromultimeric immunogens of undefined composition (Fig. 1*A*). Specifically, the coexpressed Fc fused RBD_1_ and RBD_Wu_ are expected to generate mixtures of homo- and heterodimers, while the juxtaposition of RBD_1_ and RBD_Wu_ on a mixed LzS nanoparticle will inevitably be heterogeneous. These properties might reduce the ability of the heteromultimeric immunogen to selectively cross-link B cell receptors that cross-react with both of the RBD components and thus reduce the selective expansion of cross-reactive B cells. Therefore, we focused our attention on the RBD_1_-RBD_Wu_ fusion dimers, where the composition and juxtaposition of RBD_1_ and RBD_Wu_ are defined (Fig. 1*A*).

**Fig. 2.**
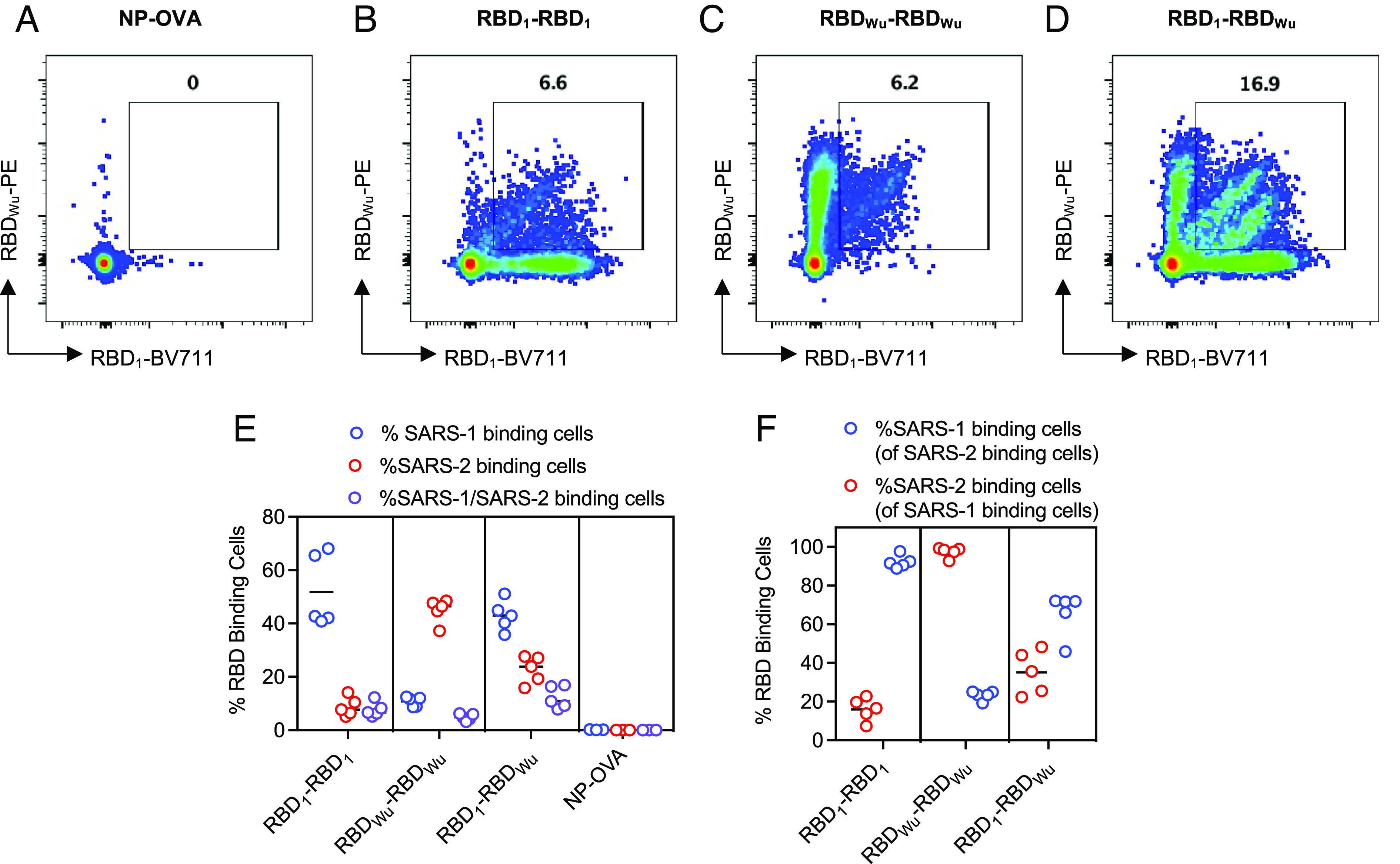
Cross-reactive B cell response to RBD homodimers and heterodimers. (*A*–*D*) Examples of FACS analysis of mouse popliteal lymph node GC B cells (B220^+^, CD4^−^, CD8^−^, Ly-6G/Ly-6C^−^, NK1.1^−^, F4/80^−^, CD38^−^, and CD95^+^) binding to fluorophore-conjugated RBD_1_ and RBD_Wu_. Mice were footpad immunized with NP-OVA as a control (*A*), or RBD_1_-RBD_1_ (*B*) RBD_Wu_-RBD_Wu_ (*C*) or RBD_1_-RBD_Wu_ (*D*). (*E*) Percentage of popliteal GC B cells that bound RBD_1_, RBD_Wu_, or both following the indicted immunizations. (*F*) Percentage of GC B cells that bound one RBD_1_, or RBD_Wu_ that also bound the other RBD following immunization with the homodimers or heterodimer (*F*). For (*E*) and (*F*) each symbol = 1 mouse, lines group mean, n = 5 mice.

To compare the extent to which the RBD homodimers and heterodimers could elicit RBD_1_-specific, RBD_Wu_-specific or cross-reactive B cells, we immunized mice by footpad injection with RBD homodimers, heterodimer or an NP-OVA control and stained germinal center (GC) B cells (B220^+^, CD4^−^, CD8^−^, Ly-6G/Ly-6C^−^, NK1.1^−^, F4/80^−^, CD38^−^, CD95^+^) isolated from the draining popliteal lymph node with fluorescently labeled RBD_1_ and RBD_Wu_ proteins (Fig. 2 *A*–*F* and *SI Appendix*, Fig. S2 *A*–*D*). The RBD_1_-RBD_1_ and RBD_Wu_-RBD_Wu_ homodimers generated a mean±sd of 52±14% and 45±5% of GC B cells, respectively, that bound to the homologous RBD (Fig. 2 *B*, *C*, and *E*), but only 8 ± 3% and 5 ± 1% GC B cells that bound to both RBD_1_ and RBD_Wu_ (Fig. 2 *B*, *C*, and *E*). The RBD_1_-RBD_Wu_ heterodimers generated 43 ± 14% RBD_1_-binding, 23 ± 6% RBD_Wu_-binding and 12±4% cross-reactive GC B cells (Fig. 2 *D* and *E*). If only RBD-binding B cells were considered, for the RBD_1_-RBD_1_ and RBD_Wu_-RBD_Wu_ immunogens, 16 ± 6% and 23 ± 2% respectively of the homologous RBD binding GC B cells also bound the heterologous RBD (Fig. 2*F* and *SI Appendix*, Fig. S2 *B* and *C*) while for the RBD_1_-RBD_Wu_ heterodimer 35±11% of the RBD_1_-binding B cells also bound RBD_Wu_ and 65±11% of the RBD_Wu_-binding B cells also bound RBD_1_ (Fig. 2*F* and *SI Appendix*, Fig. S2*D*). Overall, the RBD_1_-RBD_Wu_ heterodimer elicited a greater fraction of cross-reactive GC B cells than did the homodimers.

### SARS-CoV and SARS-CoV-2 RBD Cross-Reactive Antibody Binding.

**Fig. 3.**
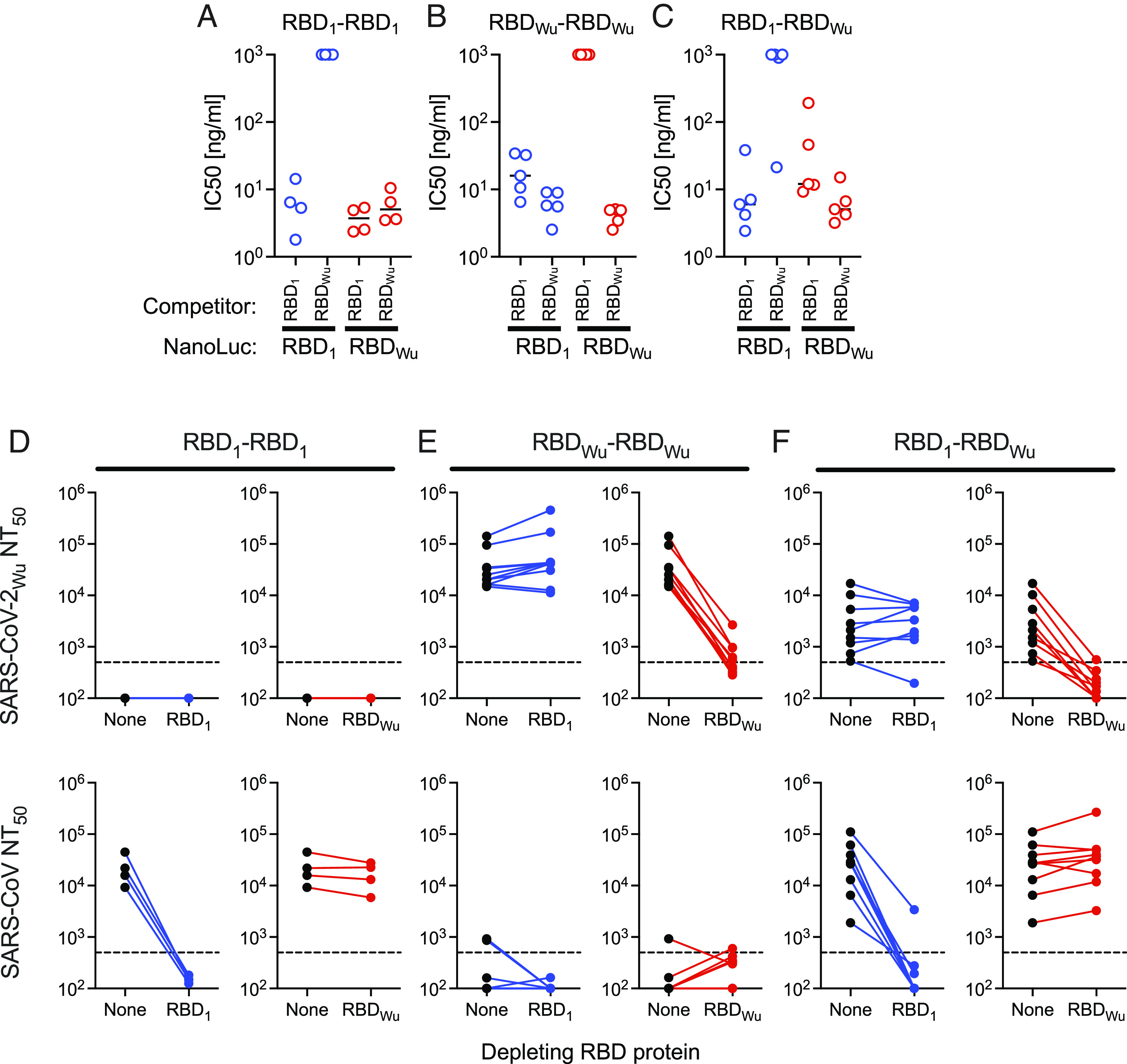
RBD_1_ and RBD_Wu_ cross-reactive serum antibodies in homodimer and heterodimer immunized mice. (*A*–*C*) Fifty percent Inhibitory concentration values (IC_50_) for RBD_1_ and RBD_Wu_ in homologous or heterologous competition assays with RBD_1_-NanoLuc and RBD_Wu_-NanoLuc. The unfused and NanoLuc-fused RBD proteins compete for binding to serum antibodies from mice immunized with RBD_1_-RBD_1_ (*A*), RBD_Wu_-RBD_Wu_ (*B*) or RBD_1_-RBD_Wu_ (*C*). Antibody-RBD/RBD-NanoLuc complexes in solution were captured with protein-G Dynabeads and bound NanoLuc activity measured. Each symbol = 1 mouse, lines = group mean, n = 5 mice. (*D*–*F*) Neutralizing antibody titers (NT_50_) against SARS-CoV-2_Wu_ (*Upper*) and SARS-CoV (*Lower*) in sera from mice immunized with RBD_1_-RBD_1_ (*D*), RBD_Wu_-RBD_Wu_ (*E*) or RBD_1_-RBD_Wu_ (*F*) following mock depletion (None) or depletion with the indicated RBD protein Each symbol = 1 mouse. The dotted line indicates the lowest serum dilution tested (1:500).

To assess the degree to which the RBD-binding serum antibodies elicited by homodimers and heterodimers were cross-reactive with both RBD_1_ and RBD_Wu_, we established a competition binding assay (*SI Appendix*, Fig. S3 *A*–*C*). RBD proteins were expressed as a fusion with NanoLuc luciferase, and each of the RBD-NanoLuc fusion proteins (*SI Appendix*, Fig. S3*A*) was incubated with serum from mice immunized with RBD_1_-RBD_1_, RBD_Wu_-RBD_Wu_, and RBD_1_-RBD_Wu_ beginning 11 to 12 wk previously. Then, the antibody:RBD-NanoLuc complexes were magnetically separated with protein G Dynabeads (*SI Appendix*, Fig. S3*B*), and the RBD_1_-NanoLuc and RBD_Wu_-NanoLuc binding capacity of each mouse serum was determined in the absence of competitor (*SI Appendix*, Fig. S3*C*). Thereafter, serum volumes containing equivalent amounts of RBD-NanoLuc binding antibody were simultaneously incubated with a fixed, saturating concentration of RBD-NanoLuc (10 ng) along with a variable (0.1× to 50×) concentration of an unlabelled RBD_1_ or RBD_Wu_ competitor (*SI Appendix*, Figs. S3*B* and S4 and Fig. 3 *A*–*C*). As expected, homologous competition binding assays (RBD_1_ competition with RBD_1_-NanoLuc and RBD_Wu_ competition with RBD_Wu_-NanoLuc) yielded mean IC_50_ values of 4.2 ± 1.1 to 19.9 ± 2.7 ng/mL, regardless of whether animals were immunized with RBD_1_-RBD_1_, RBD_Wu_-RBD_Wu_, and RBD_1_-RBD_Wu_ (Fig. 3 *A*–*C* and *SI Appendix*, Fig. S4). Conversely, heterologous binding competition assays (RBD_Wu_ competition with RBD_1_-NanoLuc and RBD_1_ competition with RBD_Wu_-NanoLuc) yielded results that were highly dependent on the immunogen. In RBD_1_-RBD_1_ immunized mice, RBD_1_ competed with RBD_Wu_-NanoLuc (mean IC_50_ = 3.8 ± 1.6 ng/mL) but RBD_Wu_ did not compete with RBD_1_-NanoLuc (mean IC_50_ > 1,000 ng/mL) (Fig. 3*A* and *SI Appendix*, Fig. S4). In RBD_Wu_-RBD_Wu_ immunized mice, RBD_1_ did not compete with RBD_Wu_-NanoLuc (mean IC_50_ = >1000 ng/mL) but RBD_Wu_ competed with RBD_1_-NanoLuc (mean IC_50_ = 6.4 ± 2.7 ng/mL) (Fig. 3*B* and *SI Appendix*, Fig. S4). These results suggest that even though RBD_1_ and RBD_Wu_ share 78% sequence identity (*SI Appendix*, Fig. S1*C*, the majority of RBD binding serum antibodies elicited by RBD_1_-RBD_1_ or RBD_Wu_-RBD_Wu_ homodimers were not cross-reactive with the heterologous RBD.

In mice immunized with RBD_1_-RBD_Wu_ heterodimers, there was competition between unfused RBD and heterologous RBD-NanoLuc serum antibodies that was variable among individual mice. Specifically, RBD_1_ competed for RBD_Wu_-NanoLuc binding (mean IC_50_ = 54 ± 79 ng/mL) and in one of five mice, and RBD_Wu_ competed for RBD_1_-NanoLuc binding (mean IC_50_ = 784 ± 429 ng/mL) (Fig. 3*C* and *SI Appendix*, Fig. S4). Thus, the heterodimeric RBD_1_-RBD_Wu_ immunogen exhibited a greater propensity to elicit cross-reactive antibodies that bound to both RBD_1_ and RBD_Wu_ than did either homodimer.

### SARS-CoV and SARS-CoV-2 Cross-Reactive Neutralizing Antibodies.

We next tested whether antibodies elicited by RBD homodimers or heterodimers exhibited cross-neutralizing activity. We incubated serum from RBD dimer-immunized mice with RBD_1_-6xHis or RBD_Wu_-6xHis tagged proteins, depleted RBD-antibody complexes with His-Tag Dynabeads and measured residual neutralizing activity against pseudotyped viruses (Fig. 3 *D*–*F*). SARS-CoV neutralizing antibodies (mean NT_50_) from RBD_1_-RBD_1_ immunized mice were reduced from 22,915 ± 15,437 to below the level of detection (<500) following depletion with RBD_1_-6xHis, but were not reduced by RBD_Wu_-6xHis depletion (mean NT_50_ = 17,284 ± 9,700) (Fig. 3*D*). Similarly, SARS-CoV-2 _Wu_ neutralizing antibodies from RBD_Wu_-RBD_Wu_ immunized mice (mean NT_50_ = 41,859 ± 42,345) were depleted by RBD_Wu_-6xHis (mean NT_50_ = 750 ± 719) but not by RBD_1_-6xHis (mean NT_50_ = 89,431 ± 135,929) (Fig. 3*E*).

Notably, the RBD_1_-RBD_Wu_ heterodimer elicited SARS-CoV neutralizing antibodies (mean NT_50_ = 35,042 ± 33,546) that were reduced by RBD_1_-6xHis depletion (to NT_50_= 498 ± 1,093) but not by RBD_Wu_-6xHis depletion (mean NT_50_ = 56,548 ± 80,861) (Fig. 3*F*). Similarly, SARS-CoV-2 neutralizing antibodies elicited by the RBD_1_-RBD_Wu_ heterodimer (mean NT_50_ = 4,630 ± 5,597) were reduced by RBD_Wu_-6xHis depletion (mean NT_50_ < 500) but not by RBD_1_-6xHis depletion (NT_50_ = 3,792 ± 2,634) (Fig. 3*F*). Thus, the RBD_1_-RBD_Wu_ heterodimer elicited polyclonal antibodies that neutralized SARS-CoV and SARS-CoV-2. However, each neutralizing activity was mediated by largely separate groups of antibodies, not by cross-reactive antibodies that bound to both RBDs.

### Antibodies Elicited by SARS-CoV-2 Variant Homodimers and Heterodimers.

We next constructed RBD homodimers and heterodimers based on SARS-CoV-2 variants that are more closely related to each other than SARS-CoV and SARS-CoV-2_Wu_ are (*SI Appendix*, Fig. S1). Specifically, we chose SARS-CoV-2_Wu_ and SARS-CoV-2_BA.5_, whose RBDs share 93% amino acid identity, but differ in susceptibility to many (but not all) SARS-CoV-2 neutralizing monoclonal antibodies (7). We immunized groups of mice with RBD_Wu_-RBD_Wu_ or RBD_BA.5_-RBD_BA.5_ homodimers or RBD_Wu_-RBD_BA.5_ heterodimers. Then, using sera collected 11 to 12 wk after the first immunogen injection, we performed the RBD:RBD-NanoLuc fusion protein competition binding and RBD-6xHis-Dynabead neutralization-depletion assays to determine the extent to which homodimer and heterodimer immunized mice generated RBD_Wu_ and RBD_BA.5_ cross-reactive binding and neutralizing antibodies.

**Fig. 4.**
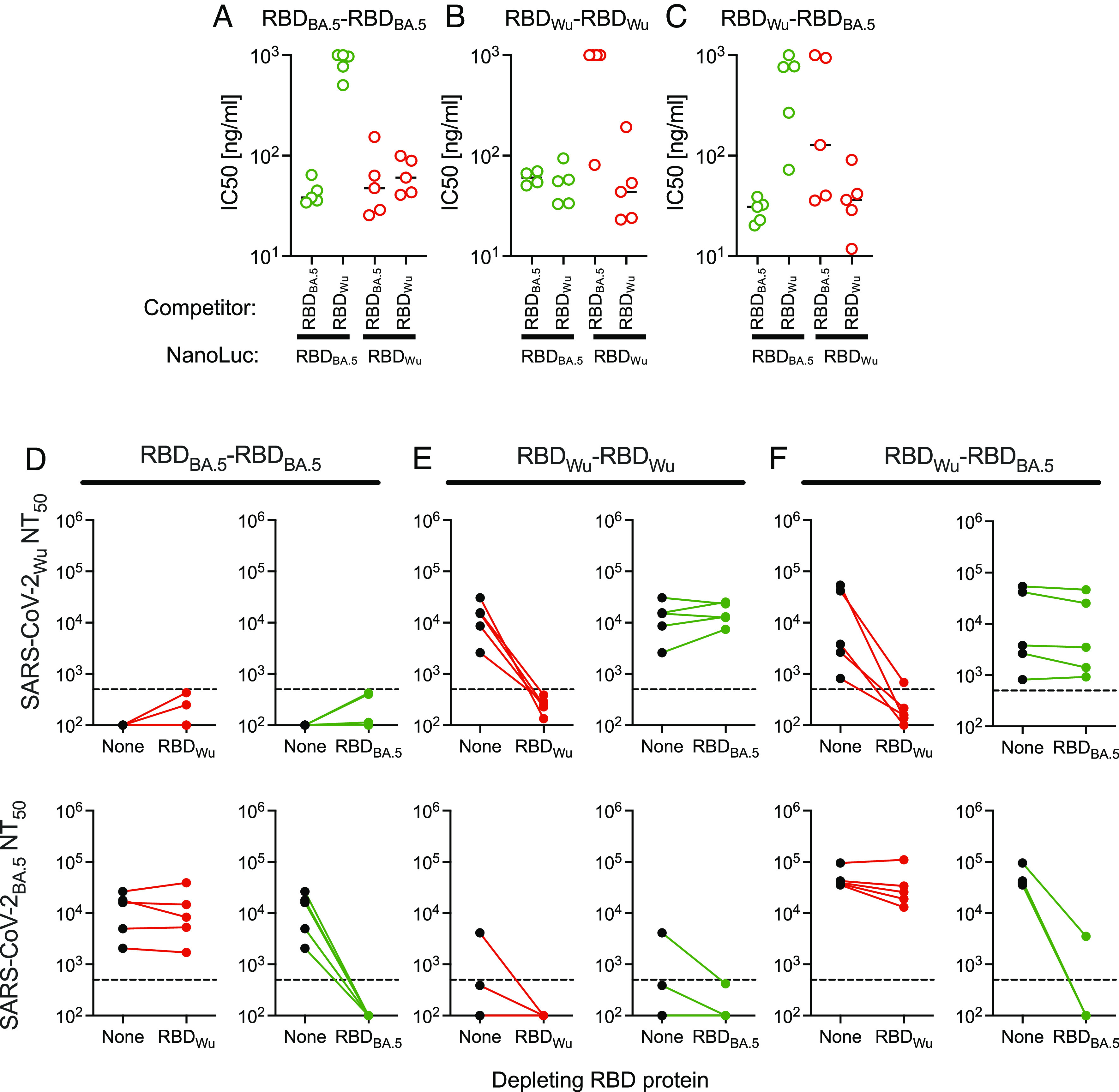
RBD_BA.5_ and RBD_Wu_ cross-reactive serum antibodies in homodimer and heterodimer immunized mice. (*A*–*C*) Fifty percent inhibitory concentration values (IC_50_) for RBD _BA.5_ and RBD_Wu_ in homologous or heterologous competition binding assays with RBD_BA.5_-NanoLuc and RBD_Wu_-NanoLuc. The unfused and NanoLuc-fused RBD proteins compete for binding to serum antibodies from mice immunized with RBD_BA.5_-RBD_BA.5_ (*A*), RBD_Wu_-RBD_Wu_ (*B*) or RBD_Wu_-RBD_BA.5_ (*C*). Antibody-RBD/RBD-NanoLuc complexes in solution were captured with protein-G Dynabeads and bound NanoLuc activity measured. Each symbol = 1 mouse, lines = group mean, n = 5 mice. (*D*–*F*) Neutralizing antibody titers (NT_50_) against SARS-CoV-2_Wu_ (*Upper*) and SARS-CoV-2_BA.5_ (*Lower*) in sera from mice immunized with RBD_BA.5_-RBD_BA.5_ (*D*), RBD_Wu_-RBD_Wu_ (*E*), or RBD_Wu_-RBD_BA.5_ (*F*) following mock depletion (None) or depletion with the indicated RBD protein. Each symbol = 1 mouse. The dotted line indicates the lowest serum dilution tested (1:500).

In assays where homologous binding competition (RBD_BA.5_ competition with RBD_BA.5_-NanoLuc and RBD_Wu_ competition with RBD_Wu_-NanoLuc) was measured, IC_50_ values of 29.0 ± 7.6 to 67.3 ± 71.2 ng/mL, were obtained and were similar whether animals were immunized with RBD _BA.5_-RBD_BA.5_, RBD_Wu_-RBD_Wu_, or RBD_Wu_-RBD_BA.5_ (Fig. 4 *A*–*C* and *SI Appendix*, Fig. S5). Conversely, in heterologous binding competition assays (RBD_Wu_ competition with RBD_BA.5_-NanoLuc and RBD_BA.5_ competition with RBD_Wu_-NanoLuc), results varied with the immunogen. In RBD_BA.5_-RBD_BA.5_ immunized mice, RBD_BA.5_ competed with RBD_Wu_-NanoLuc (mean IC_50_ = 63.7 ± 52.5 ng/mL), but RBD_Wu_ competed less well with RBD_BA.5_-NanoLuc (mean IC_50_ = 850±215 ng/mL) (Fig. 4*A* and *SI Appendix*, Fig. S5). In sera from RBD_Wu_-RBD_Wu_ immunized mice, only 2/5 mice exhibited detectable RBD_BA.5_ competition with RBD_Wu_-NanoLuc (mean IC_50_ = 816 ± 411 ng/mL) but RBD_Wu_ competed with RBD_BA.5_-NanoLuc (mean IC_50_ = 54.7 ± 24.9 ng/mL) (Fig. 4*B* and *SI Appendix*, Fig. S5). Overall, in RBD_Wu_ and RBD_BA.5_ homodimer immunized mice, most RBD-binding serum antibodies were specific for the homologous RBD, but a small proportion of antibodies also bound to the heterologous RBD.

In sera from mice immunized with RBD_Wu_-RBD_BA.5_ heterodimers, competition for antibody binding between heterologous RBDs was evident in sera from some but not all mice. Overall, RBD_BA.5_ competed for RBD_Wu_-NanoLuc binding (mean IC_50_ = 429 ± 496 ng/mL) and RBD_Wu_ competed for RBD_BA.5_-NanoLuc binding (mean IC_50_ = 576 ± 388 ng/mL) (Fig. 4*C* and *SI Appendix*, Fig. S5). We conclude that the RBD_Wu_-RBD_BA.5_ heterodimer exhibited greater propensity to elicit cross-reactive RBD binding antibodies than did either of the two homodimers.

We next used the RBD-6xHis-Dynabead depletion assay to measure whether neutralizing antibodies elicited by RBD_Wu_ and RBD_BA.5_ homodimers or heterodimers were cross-reactive. SARS-CoV-2_BA.5_ neutralizing antibodies from RBD_BA.5_-RBD_BA.5_, immunized mice were reduced from (mean ± SD NT_50_ = 13,417 ± 9,894) to below the level of detection (<500) following depletion with RBD_BA.5_-6xHis but were not reduced by RBD_Wu_-6xHis depletion (mean NT_50_ = 13,804 ± 14,875) (Fig. 4*D*). Similarly, SARS-CoV-2_Wu_ neutralizing antibodies from RBD_Wu_-RBD_Wu_ immunized mice (mean NT_50_ = 14,497 ± 10,456) were reduced by RBD_Wu_-6xHis depletion (mean NT_50_ < 500) but not by RBD_BA.5_-6xHis depletion (mean NT50 = 16,336 ± 7,652) (Fig. 4*E*).

The RBD_Wu_-RBD_BA.5_ heterodimer elicited SARS-CoV-2_BA.5_ neutralizing antibodies (mean NT_50_ = 49,833 ± 25,504) that were depleted by RBD_BA.5_-6xHis (to NT_50_ = 782 ± 1,526) but not by RBD_Wu_-6xHis depletion (NT_50_ = 40,221 ± 39,623) (Fig. 4*F*). Similarly, SARS-CoV-2_Wu_ neutralizing antibodies elicited by the RBD_Wu_-RBD_BA.5_ heterodimer (mean NT_50_ = 20,601 ± 25,289) were depleted by RBD_Wu_-6xHis depletion (mean NT_50_ < 500) but not by RBD_BA.5_-6xHis (NT_50_ = 15,506 ± 20,042) (Fig. 4*F*). Thus, as was the case for the RBD_1_-RBD_Wu_ heterodimer, the RBD_Wu_-RBD_BA.5_ heterodimer generated largely separate pools of neutralizing antibodies that were effective against SARS-CoV-2_Wu_ or SARS-CoV-2_BA.5_, but not both.

We conducted a similar experiment using a heterodimer composed of RBD_Wu_ and RBD_BA.1_. Unfortunately, the yield of the RBD_BA.1_-6xHis monomer was poor and insufficient to complete depletion experiments. Nevertheless, mice immunized with an RBD_Wu_-RBD_BA.1_ heterodimer generated neutralizing antibodies against SARS-CoV-2_Wu_ (mean NT_50_ = 5,933 ± 5,852) and SARS-CoV-2_BA.1_ (mean NT_50_ = 32,527 ± 34,410) (*SI Appendix*, Fig. S6). SARS-CoV-2_Wu_ neutralizing antibodies were depleted (mean NT_50_ = 523 ± 297) by RBD_Wu_-6His while neutralizing antibodies against SARS-CoV-2_BA.1_ were not (mean NT_50_ = 21,362 ± 28,510) (*SI Appendix*, Fig. S6).

### Long-Term Heteromultimeric RBD Exposure Does Not Broaden Serum Neutralizing Antibody Specificity.

The neutralization breadth of individual SARS-CoV-2 antibodies in the memory B cell compartment following infection or vaccination increases with time, as a consequence of ongoing somatic mutation, presumably driven by residual persisting antigen (20–24). In principle, therefore, restimulation of B cells by persistent, heterogenous, antigen exposure might broaden the specificity of serum antibodies through the selective expansion and affinity maturation of B cells that acquire the ability to react with multiple components of a heteromultimeric antigen.

To expose B cells to with heterogeneously juxtaposed, heteromultimeric RBD antigens for prolonged periods, we constructed recombinant adeno-associated virus vectors (rAAVs) expressing RBD-LzS nanoparticles. Immunization of mice with a single intramuscular dose of an rAAV expressing RBD_Wu_-LzS, but not control vector expressing mNeonGreen, generated similar titers of SARS-CoV-2_Wu_ neutralizing antibodies (mean ± SD NT_50_ = 33,040 ± 22,254) as did two doses (at week 0 and week 3) of the corresponding recombinant RBD_Wu_-LzS protein with adjuvant (mean NT_50_ = 48,497 ± 21,360) (*SI Appendix*, Fig. S7*A*). These neutralizing titers were measured at 7 to 8 wk after initial immunization, at approximately the time they reached a plateau for both immunization approaches. The rAAV immunized mice were maintained for a total of ~6 mo and neutralizing titers remained approximately constant at this level (*SI Appendix*, Fig. S7*A*).

**Fig. 5.**
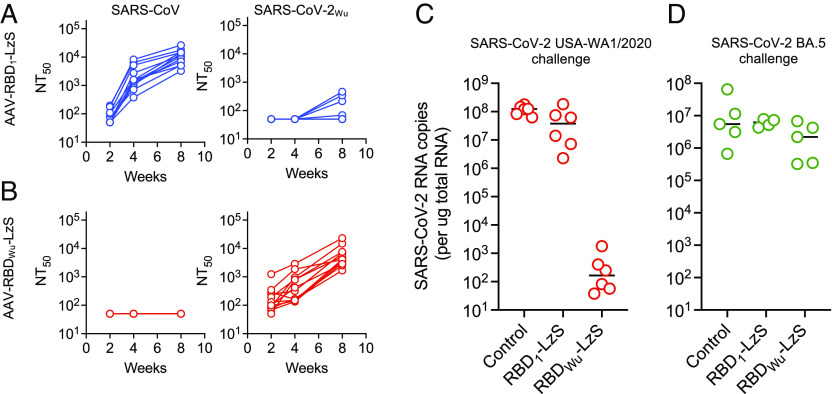
Neutralizing antibodies and SARS-CoV-2 variant specific protection by single-dose rAAV RBD-LzS immunization. (*A*) Neutralizing titers (NT_50_) against SARS-CoV and SARS-CoV-2_Wu_ pseudotypes over 8 wk following immunization with a single dose of rAAV RBD_1_-LzS (*A*) or rAAV RBD_Wu_-LzS (*B*). (*C* and *D*) Lung viral load (SARS-CoV-2 RNA copies per μg total RNA) at day 3 after challenge in mice following immunization with rAAV RBD_1_-LzS or rAAV RBD_Wu_-LzS as indicated and challenge with SARS-CoV-2 USA-WA1/2020 (*C*) or SARS-CoV-2 BA.5 (*D*) at 8 wk after immunization. Each symbol = 1 mouse, lines = group mean, n = 6 mice per group.

As was the case with recombinant RBD proteins (Fig. 3), neutralizing antibodies elicited by rAAV vectors expressing RBD_1_-LzS or RBD_Wu_-LzS at 8 wk after administration, were largely specific for the corresponding virus pseudotype (Fig. 5 *A* and *B*). Control rAAV immunizations elicited no pseudotype neutralizing activity (*SI Appendix*, Fig. S7*B*). Challenge of human ACE2 expressing mice (K18-hACE2) with SARS-CoV-2 USA-WA/2020, which shares an identical RBD sequence with RBD_Wu_, 8 wk after immunization with rAAV RBD_Wu_-LzS resulted in strong protection, with lung viral RNA levels reduced by >5 orders of magnitude compared to controls (*P* = 0.0009, Welch’s *t* test) (Fig. 5*C*). Conversely, immunization with rAAV RBD_1_-LzS gave a nonsignificant (*P* = 0.09) reduction in lung SARS-CoV-2 USA-WA/2020 load. Infection of similarly rAAV RBD_1_-LzS or rAAV RBD_Wu_- LzS immunized mice with SARS-CoV-2_BA.5_ gave no discernable reduction in viral load (Fig. 5*D*). Thus, a single injection of a single rAAV RBD-LzS immunogen generated strongly protective neutralizing antibodies against the homologous virus, but these antibodies lacked sufficient breadth to confer protection against a heterologous virus at 8 wk after immunization.

To attempt to broaden the neutralizing antibody response elicited by rAAV-RBD_1_-LzS and rAAV-RBD_Wu_-LzS, we generated a scenario in which mouse B cells would be exposed, over an extended period, to heteromultimeric antigen in the form of LzS particles bearing a mixture of RBD_1_ and RBD_Wu_. To generate mixed RBD particles, individual cells would need to be transduced with both rAAV RBD_1_-LzS and rAAV RBD_Wu_-LzS. To test whether this would be the case, we administered the same dose of a single rAAV encoding either mNeonGreen or mScarlet-I fluorescent proteins, or a mixture of the two to a single hind limb muscle, and then used microscopy to determine whether myotubes expressed either or both fluorescent reporters. In coinjected mice, all of the imaged myotubes expressed both mNeonGreen and mScarlet-I, indicating that they received both constituents of a mixed, 2-component rAAV-injection (*SI Appendix*, Fig. S8*A*). Moreover, rAAV-driven expression of both mNeonGreen and mScarlet-I was maintained in myotubes for at least 6 mo (*SI Appendix*, Fig. S8*B*).

We next injected mice with equivalent doses of rAAV RBD_1_-LzS, rAAV RBD_Wu_-LzS, or both to generate nanoparticles bearing a single RBD-LzS protein or a mixture of RBD_1_-LzS and RBD_Wu_-LzS. We used the RBD-6xHis-Dynabead depletion assay to determine whether neutralizing antibodies elicited by the nanoparticles at 10 to 12 wk after rAAV injection were cross-reactive. Sera from rAAV RBD_1_-LzS immunized mice neutralized SARS-CoV (mean ± SD NT_50_ = 8,135 ± 5,041) but not SARS-CoV-2_Wu_ and this neutralizing activity was depleted by RBD_1_-6xHis (mean NT_50_ < 500) but not by RBD_Wu_-6xHis (mean NT_50_ = 10,364 ± 5,060) (*SI Appendix*, Fig. S9*A*). Conversely, sera from rAAV RBD_Wu_-LzS immunized mice neutralized SARS-CoV-2 (mean ± SD NT_50_ = 5,509 ± 3,619) but not SARS-CoV and this neutralizing activity was depleted by RBD_Wu_-6xHis (mean NT_50_ < 500) but not by RBD_1_-6xHis (mean NT_50_ = 3,474 ± 2,653) (*SI Appendix*, Fig. S9*B*). Mice injected with a mixture of rAAV RBD_1_-LzS and rAAV RBD_Wu_-LzS generated neutralizing antibodies against both SARS-CoV and SARS-CoV-2_Wu_ (mean NT_50_ = 1,480 ± 1,138 and 10,766 ± 9,073 respectively). SARS-CoV neutralizing activity was depleted by RBD_1_-6xHis (mean NT_50_ < 500) but not RBD_Wu_-6xHis (mean NT_50_ = 1,480 ± 1,138) while SARS-CoV-2_Wu_ neutralizing activity was depleted by RBD_Wu_-6xHis (mean NT_50_ < 500) but not RBD_1_-6xHis (mean NT_50_ = 7,049 ± 5,653) (*SI Appendix*, Fig. S9*C*). Notably, mixing rAAV RBD_1_-LzS and rAAV RBD_Wu_-LzS prior to administration in a single hind limb muscle (*SI Appendix*, Fig. S9*C*) did not elicit greater breadth than administration of the same doses of rAAV RBD_1_-LzS and rAAV RBD_Wu_-LzS separately in left and right hind limb muscles (*SI Appendix*, Fig. S9*D*).

**Fig. 6.**
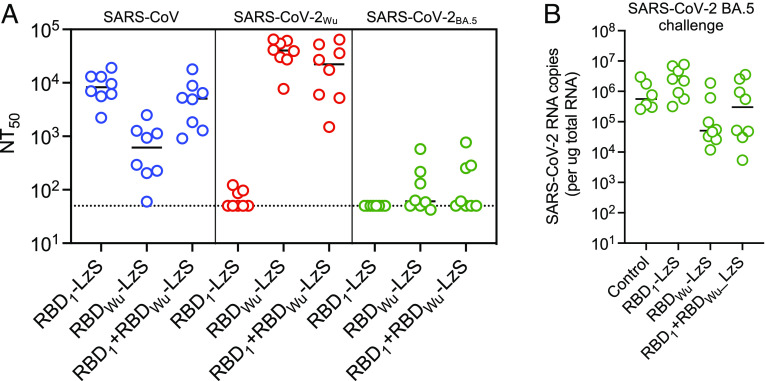
Neutralizing antibodies and heterologous challenge 24 wk after rAAV RBD-LzS immunization. (*A*) Neutralizing titers (NT_50_) against SARS-CoV, SARS-CoV-2_Wu_, and SARS-CoV-2_BA.5_ pseudotypes at 24 wk after immunization with a single dose of rAAV RBD_1_-LzS, rAAV RBD_Wu_-LzS or a mixture of both in a single injection. The dotted line indicates the lowest sera dilution tested (1:50). (*B*) Lung viral load (SARS-CoV-2 RNA copies per μg total RNA) at day 3 after challenge in mice following immunization with rAAV RBD_1_-LzS, rAAV RBD_Wu_-LzS or a mixture of both in a single injection, as indicated, and challenge at 24 wk after immunization with SARS-CoV-2 BA.5 Each symbol = 1 mouse, lines = group mean, n = 8 mice.

Next, we injected K18 hACE2 mice with the same doses of rAAV RBD_1_-LzS, rAAV RBD_Wu_-LzS, or both, as a mixture in a single hind limb and maintained them for 24 wk to allow an extended period of B cell and antibody evolution before challenge with a heterologous virus, SARS-CoV-2_BA.5_. In mice immunized with rAAV RBD_1_-LzS alone, SARS-CoV neutralizing antibodies were generated that persisted for at least 24 wk (mean ± SD NT_50_ = 9,480 ± 5,398 at week 24) accompanied by low titers against SARS-CoV-2 (mean NT_50_ = 69 ± 29) (Fig. 6*A* and *SI Appendix*, Fig. S10*A*). Antibodies against SARS-CoV-2_BA.5_ were undetectable (NT_50_ < 50). In rAAV RBD_Wu_-LzS immunized mice, high titers of SARS-CoV-2_Wu_- neutralizing antibodies (mean NT_50_ = 40,790 ± 19,173 at week 24) and low titers of SARS-CoV neutralizing antibodies (mean NT_50_ = 825 ± 817) were elicited (Fig. 6*A* and *SI Appendix*, Fig. S10*B*). In mice immunized with both rAAV RBD_1_-LzS and rAAV RBD_Wu_-LzS neutralizing antibodies against both SARS-CoV and SARS-CoV-2_Wu_ (mean NT_50_ = 5,929 ± 5,598 and 26,226 ± 23,215, respectively at 24 wk) were generated (Fig. 6*A* and *SI Appendix*, Fig. S10*C*).

Some neutralizing activity against SARS-CoV2_BA.5_ was acquired in mice immunized with rAAV RBD_Wu_-LzS alone and in mice immunized with both rAAV RBD_1_-LzS and rAAV RBD_Wu_-LzS (Fig. 5*A* and *SI Appendix*, Fig. S10 *B* and *C*). This neutralizing activity was acquired at later time points, after titers against SARS-CoV-2_Wu_ and SARS-CoV had reached a plateau, suggesting a role for extended B cell evolution in its generation. However, coadministration of rAAV RBD_1_-LzS with rAAV RBD_Wu_- LzS to generate the mixed nanoparticles did not result in increased titers against SARS-CoV-2_BA.5_ (mean NT_50_ = 196 ± 256) compared to immunization with rAAV RBD_Wu_- LzS alone (mean NT_50_ = 149 ± 183, *P* = 0.856) (Fig. 6*A* and *SI Appendix*, Fig. S10 *B* and *C*). Moreover, challenge of rAAV RBD_1_- LzS + rAAV RBD_Wu_- LzS immunized K18-hACE2 mice with SARS-CoV-2_BA.5_ did not result in reduced viral load compared to mice immunized with rAAV RBD_Wu_-LzS alone (*P* = 0.27) or nonimmunized controls (*P* = 0.88) (Fig. 6*B*).

Overall, these data suggest that coadministration of rAAV RBD_1_-LzS with rAAV RBD_Wu_-LzS generated largely separate sets of SARS-CoV and SARS-CoV-2_Wu_ neutralizing antibodies. A period of 12 to 24 wk of antigen exposure was accompanied with the generation of some level of antibodies capable of neutralizing a divergent virus (SARS-CoV-2_BA.5_) that was not included in the immunogen. However, these antibodies were insufficient for protection against infection and were generated at equivalent levels whether mice were subjected to extended exposure to nanoparticles bearing single heterologous RBD antigen or two diverse heterologous RBD antigens.

## Discussion

It is thought that vaccines which display multiple, physically linked but diverse sarbecovirus RBDs would selectively cross-link B cell receptors (BCRs) that bind conserved epitopes or BCRs that tolerate epitope variation. Selective BCR cross-linking in this way might selectively stimulate B cells that can go on to secrete broadly reactive antibodies (28–31). Indeed, in animals that have been immunized with mosaic RBD nanoparticles, antibodies were directed to more conserved epitopes (30) and individual broadly neutralizing antibodies can be generated (34). However, in a polyclonal response to physically linked heterologous RBDs, it is unclear what fraction of antibodies cross-react with multiple RBDs and what fraction are specific to a single RBD component. Similarly, multivalent mRNA spike vaccines such as the Wuhan-hu-1/BA.5 mixture deployed in 2022, in which mRNAs were mixed prior to administration, would generate a mixture of homomultimeric and heteromultimeric trimers (35, 36). In a naive recipient, these trimers could, in principle, selectively stimulate cross-reactive B cells or stimulate separate Wuhan-hu-1-specific and BA.5-specific neutralizing antibody generating cells.

We found that multimerized RBD immunogens containing a single ancestral SARS-CoV-2 RBD species generated higher serum neutralizing antibody titers than the equivalent monomeric RBD. The superiority of the multimerized RBD in the generation of high serum neutralizing activity was evident irrespective of whether multimerization was mediated by genetic fusion to a second RBD in a single polypeptide, fusion to IgG1 Fc that spontaneously dimerizes, or fusion to LzS that spontaneously assembles into nanoparticles. This finding is consistent with the idea that artificial multimerization augments stimulation of naive B cells through BCR cross-linking (37). Analysis of the RBD binding specificity of germinal center B cells and selective depletion of RBD binding antibodies in serum generated using genetically fused SARS-CoV-2_Wu_ RBD and either SARS-CoV or SARS-CoV-2_BA.5_ RBD suggests that heterodimeric immunogens can bias the response, to some degree, toward B cells and serum antibodies that bind both components of a heterodimer and are therefore cross-reactive. Again, this is consistent with the notion that a multimeric immunogen is better able to cross-link cross-reactive BCRs the depletion with one antigen, and heterodimeric antigens can elicit antibodies that cross-react with both components.

However, when antibodies from RBD heterodimer-immunized mice that bound one of the two RBD components were depleted from serum, neutralizing activity against the homologous viral pseudotype was removed, but neutralization activity against pseudotypes corresponding the other dimer component was unaffected. Thus, the polyclonal antibody pool generated by RBD heterodimers appears to contain two sets of neutralizing antibodies that are almost entirely separate and does not consist of cross-reactive antibodies. Notably, the SARS-CoV and SARS-CoV-2_BA.5_ RBD amino acid sequences are 78% and 93% identical to the SARS-CoV-2_Wu_ RBD sequence respectively, but variation is concentrated in the neutralizing epitopes (12, 17). Greater divergence in the neutralizing epitopes of an RBD heteromultimer would reduce the probability that the subset of naive BCRs that can be cross-linked by heteromultimers represent those that recognize neutralizing epitopes and therefore can go on to generate neutralizing antibodies. Thus, the initial B cell response to a heteromultimeric RBD immunogen may be biased toward conserved, but mostly non-neutralizing, epitopes.

Naive BCRs that are capable of reacting with two different neutralizing epitopes on two components of an RBD heteromultimer may be extremely rare. However, individual SARS-CoV-2 RBD-binding neutralizing antibodies that appear in the memory B cell compartment acquire diversity and greater affinity in the months following infection or vaccination (20–24). In some cases, this maturation enables recognition and neutralization of sarbecoviruses that were not neutralized by the earlier antibodies and are divergent from any antigen to which the subject has been exposed (20–22, 24, 25). This property presumably reflects the evolution of BCRs in long-lived germinal centers (38, 39) and is evident to a greater degree following SARS-CoV-2 infection, where antigen may be more persistent (20, 40) than following vaccination (23). We used rAAVs to prolong exposure of the murine immune system to heteromultimeric RBD antigens in an attempt to drive affinity maturation that might broaden the reactivity of BCRs and serum antibodies. While there was some evidence that some neutralization breadth was acquired over months in mice with exposure to a persistent antigen, this effect was not greater in mice immunized with heterodimers compared to homodimers. In alternative “prime boost” strategy, animals might be first immunized with one antigen component to generated diversified BCRs that bind one antigen and then boosted with a second variant to selectively expand cross-reactive antibodies. Whether such an approach is better able to generate cross-neutralizing antibodies than prolonged exposure to heterodimers will require further experimentation.

Overall, while heteromultimeric RBD immunogens may bias a murine B cell response toward cross-reactive antibodies to some extent, the results herein suggest that simply combining divergent RBDs results in a B cell response consisting of largely separate sets of single RBD-specific neutralizing serum antibodies. These antibodies are mostly incapable of neutralizing viruses that diverge from the immunogen components. Careful consideration of the genetic and antigenic distance between the components of multimeric immunogen-based vaccines may be required to generate antibodies with maximum possible neutralizing breadth, and particularly with activity against viruses whose sequences differ substantially from the immunogen components. Such considerations are of key importance when designing vaccines in the context of an evolving virus such as SARS-CoV-2 and in preparation for potential future viral pandemics.

## Materials and Methods

### RBD Expression Plasmids.

Synthetic DNA sequences encoding the RBD of SARS-CoV-2 (Wuhan-hu-1 variant), termed RBD_Wu_, human IgG1Fc, and Lumazine Synthase were purchased from GeneArt (ThermoFisher). Sequences encoding the RBD of SARS-CoV (RBD_1_) and other SARS-CoV-2 variants (RBD_BA.1_, RBD_BA.5_) were amplified from previously described full-length spike expression plasmids (6, 7, 41). The RBD_1_, RBD_Wu_, RBD_BA.1_, RBD_BA.5_ sequences were PCR amplified to introduce a secretion signal pSecTag2 (METDTLLLWVLLLWVPGSTGD) and a 6xHisTag at the N and C termini of the RBD encoding sequences. Overlap extension PCR was used to fuse RBD_Wu_ to IgG1Fc with an N-terminal pSecTag2 sequence and a C-terminal 6xHisTag or to Lumazine Synthase with an N-terminal pSecTag2-6xHisTag. The RBD, RBD-Fc, and RBD-LzS constructs were inserted into the NcoI and XhoI sites of pCAGGS to generate the mammalian protein expression plasmids. Plasmids expressing RBD-Avi-tag fusions for generating biotinylated RBD proteins were generated in the same way except that sequences encoding an Avi-tag GLNDIFEAQKIEWHE followed by a 6xHisTag were included at the C terminus. Plasmids expressing RBD-RBD fusion protein dimers (RBD_Wu_-RBD_Wu_, RBD_1_-RBD_1_, RBD_1_-RBD_Wu_, RBD_BA.5_-RBD_BA.5_, RBD_Wu_-RBD_BA.5_, and RBD_Wu_-RBD_BA.1_) were constructing by amplifying the N-terminal RBD sequence with oligonucleotides encoding an N-terminal pSecTag2 sequence and a C-terminal glycine-serine-linker (GGSGG) incorporating a NotI site, while the and the C-terminal RBD sequence was amplified to introduce a 5′ NotI site and a C-terminal 6xHisTag. Plasmids expressing the RBD-RBD fusion dimers were built using three fragment ligation, whereby the two RBD encoding sequences(NcoI-RBD-NotI-RBD-XhoI) were inserted into the NcoI and XhoI sites of pCAGGS. To express RBD-NanoLuc fusion proteins constructs, NanoLuc encoding DNA sequences were amplified from pHIV-1_NL4-3_DEnv-NanoLuc (41) introducing 5′ NotI and 3′ 6xHisTag-XhoI sequences and inserted into pCAGGS, in the same manner as for the RBD-RBD fusion dimers but with NanoLuc in place of the C-terminal RBD.

### AAV Constructs.

Synthetic DNA sequences encoding pSecTag2, fused to RBD_1_, or RBD_Wu_ and Lumazine synthase-6xHis were amplified to introduce EcoRI and HindIII restriction sites at 5′ and 3′ ends of the amplicon. These DNA fragments were inserted into adeno-associated virus (AAV) expression vector (pAAV-MCS, VPK-410, Cell Biolabs, Inc.) at the multiple cloning site (MCS) flanked by the two inverted terminal repeats (ITRs), generating pAAV-RBD_1_-LzS and pAAV-RBD_Wu_- LzS expressing plasmids respectively. mNeonGreen and mScarlet-I fluorescent protein coding sequences were PCR amplified to introduce EcoRI and HindIII restriction sites and inserted into the AAV vector in the same way generating pAAV-mNeonGreen and pAAV-mScarlet-I. The AAV packaging plasmid, pAAV-DJ Rep-Cap expressing the recombinant capsid and AAV2 Rep proteins, and helper plasmid (pAAV-Helper) expressing the E2A, 4B, and VA RNA from adenovirus were obtained from Cell Biolabs (Cat# VPK-400-DJ).

### Cell Lines.

The cell lines 293 T (CVCL_0063), 293AAV (Cell Biolabs AAV-100), HT1080 (CVCL_0317), and HT1080/ACE2.cl14 (41) were maintained in Dulbecco’s Modified Eagles Medium (DMEM, ThermoFisher 11995065) supplemented with 10% fetal calf serum (FCS, Sigma F092) and gentamicin (Gibco 15750060) at 37 °C and 5% CO_2_. VeroE6 cells (*Chlorocebus sabaeus*; sex: female, kidney epithelial) obtained from the ATCC (CRL-1586™) and from Ralph Baric (University of North Carolina at Chapel Hill) and Caco-2 cells (*Homo sapiens*; sex: male, colon epithelial) obtained from the ATCC (HTB-37™) were cultured in DMEM supplemented with 1% nonessential amino acids (NEAA, Gibco, Cat# 11140-050) and 10% fetal bovine serum (FBS, HyClone, Cat# SH30396.03) at 37 °C and 5% CO_2_. Expi293 were maintained in Expi293 Expression Medium (ThermoFisher, A1435101) at 37 °C and 8% CO_2_. All cell lines were periodically checked for mycoplasma contamination by DAPI staining and for retrovirus contamination using reverse transcriptase assays.

### AAV Production and Purification.

Recombinant AAV vector particles containing genomes expressing RBD_1_-LzS, RBD_Wu_- LzS, mNeonGreen or mScarlet-I were produced using a modified version of previously reported protocols (42). Specifically, 8 × 10^7^ 293AAV cells were cotransfected at 80 to 90% confluency with 50 μg of a pAAV-based vector, 50 μg pAAV-DJ Rep-Cap, and 50 μg pAAV-Helper in a 5-layered multiflasks (Corning, 353144) using 1 mg/mL polyethylenimine (PEI, Polysciences Cat# 23966) at a PEI:DNA ratio of 4:1. At 18 to 24 h posttransfection, the media were changed to AAV production media; DMEM + GlutaMAX-1 (Gibco 10567-014) supplemented with 1% FCS, gentamycin, 10 mM HEPES (Gibco 15630080), 1X GlutaMAX (Gibco, 35050061), and 0.075% sodium bicarbonate (Gibco, 25080094). The supernatant was harvested at 24 h later (day 3 posttransfection) and replaced with fresh production media for another 48 h. At day 5 posttransfection, cells and supernatant were harvested and processed separately. AAV particles in the day 3 and day 5 posttransfection supernatants were precipitated overnight at 4 °C with 40% polyethylene glycol (PEG) 8000 (w/w) (Sigma-Aldrich, 895101KGF) in 2.5 M NaCl in a 4:1 ratio of supernatant to PEG 8000, centrifuged at 2,500 × g for 1 h at 4 °C and resuspended in HEPES buffer. Harvested cells were lysed in a AAV lysis buffer containing 1 M Tris-HCl, 1.5 M NaCl, and 1 M MgCl_2_ at pH 8.0, freeze-thawed four times, and treated with 50 U/mL Benzonase (Millipore E1014-25KU) and 0.3 mg/mL RNAse A (Macherey-Nagel 740505). The homogenates were then incubated with 0.5% sodium deoxycholate (Sigma Aldrich, Cat# D6750-100G) at 37 °C for 30 min and subsequently cleared of debris by centrifuging at 2,500 rpm for 10 min. AAV particles were precipitated from the cleared cell lysate overnight with a 1:4 ratio of 40% PEG/2.5 M NaCl to supernatant. Following PEG precipitation and centrifugation, the AAV particles were resuspended in 1 M HEPES buffer, combined with AAV particles precipitated from the day 3 and day 5 supernatants, and further purified by chloroform extraction (Sigma-Aldrich, Cat# C2432-500ML) and aqueous two-phase separation, using 20% ammonium sulfate (w/w) (Sigma Aldrich, Cat# A4418-1 KG) and 50% PEG 8000 (w/v). The purified AAV particles were subsequently buffer exchanged with sterile PBS using Amicon Ultra-15 centrifugal filters NMWL 50 kDa cutoff (Millipore UFC905024) and filtered using 0.22 μm polyvinylidene fluoride (PVDF) centrifugal filters (Millipore UFC30GV25).

### AAV Quantification and Analysis.

AAV capsids in purified particle preparations were disrupted by alkaline (42) lysis to release the viral DNA. The liberated viral DNA was subjected to quantitative real-time polymerase chain reaction (qRT-PCR) using Power SYBR™ Green 1 Step Kit (Applied Biosystems 4389986) and universal AAV primers (Forward: 5′-GGAACCCCTAGTGATGGAGTT-3′ and Reverse: 5′-CGGCCTCAGTGAGCGA-3′) targeting the internal terminal repeats (ITRs) (43). Serial dilutions of viral plasmid containing ITRs and the extracted viral DNA were measured using a StepOnePlus™ Real-Time PCR System and the StepOne™ Software v2.2.2 (Applied Biosystems). To assess the purity of the viral preparations, the lysed AAV capsids were separated by sodium dodecyl sulfate–polyacrylamide gel electrophoresis (SDS-PAGE), NuPAGE 4 to 12% Bis–Tris Gel (Invitrogen, ThermoFisher, Cat# NP0323BOX) and stained with GelCode Blue Stain Reagent (ThermoFisher 24590) and imaged using a LI-COR Odyssey scanner (LI-COR Biosciences). To verify AAV transduction efficiency, 1 × 10^6^ HT1080 cells were seeded overnight and incubated with AAV preparations expressing RBD_1_-LzS, RBD_Wu_- LzS, mNeonGreen or mScarlet-I at 1 × 10^8^ viral genomes (vg) per well. At 72 h posttransduction, supernatants and cells were harvested, lysed, and analyzed by western blotting for the presence of AAV capsid (VP1/VP2/VP3) RBD_1_, RBD_Wu_, and 6xHisTag as described below. Expression of mNeonGreen and mScarlet-I was detected with the EVOS_fl_ fluorescent microscope (ThermoFisher).

### Western Blotting.

Monolayers of AAV-transduced HT1080 cells and supernatants were homogenized by sonication in NuPAGE™ LDS Sample Buffer (4X) (ThermoFisher NP0008), supplemented with 2.5% beta-mercaptoethanol (Fisher Chemical, Cat# O3446I-100). Lysates were incubated at 70 °C for 10 min and separated by electrophoresis in NuPAGE 4 to 12% Bis-Tris polyacrylamide gels (ThermoFisher NP0323). After transferring proteins to a 0.45 μm nitrocellulose blotting membrane (Amersham, Cat# 10600002) using XCell II™ Blot Module (ThermoFisher) and blocking with Intercept Blocking Buffer (LI-COR Biosciences), the membranes were probed overnight at 4 °C with mouse monoclonal antibodies to SARS-COV-2 RBD (Sino Biological, 40150-T62-COV2) and 6xHisTag (ThermoFisher, MA1-135)and AAV capsid (VP1/VP2/VP3) (American Research Products 03-65158) and followed by IRDye 800CW goat anti-mouse IgG secondary antibody for 1 h (LI-COR Biosciences 92632210). The blots were imaged with the LI-COR Odyssey scanner (LI-COR Biosciences).

### Recombinant Protein Expression and Purification.

All 6xHis tagged RBD proteins, RBD-RBD fusion dimers, and other RBD-fusion proteins were produced using the Expi293 Expression System (ThermoFisher A14635) according to the manufacturer’s protocol. Briefly, 75 to 600 million Expi293 were transfected with pCAGGS-based plasmids expressing RBD, RBD-Fc, RBD-LzS, or RBD dimers, using ExpiFectamine, with the addition of Enhancer 1 and 2 at 16 to 18 h posttransfection (ThermoFisher A14524). Culture supernatants were harvested at 4 to 6 d posttransfection depending on cell viability. The supernatants were passed through 0.22 μm filters (Millipore S2GPU05RE), and 1/10 volume of 500 mM NaH_2_PO_4_, 1.5 M NaCl, and 100 mM imidazole pH 8.0 was added. The supernatants were batch bound to 1 to 2 mL Ni-NTA Agarose beads (Qiagen 30210) for 2 to 16 h at 4 °C, and the beads were collected by gravity flow into a column. Beads were washed with 10 column volumes of 50 mM NaH_2_PO_4_, 300 mM NaCl, and 20 mM imidazole pH 8.0. The proteins were then eluted in 3 column volumes of 50 mM NaH_2_PO_4_, 300 mM NaCl, and 250 mM imidazole pH 8.0. Purified fractions were dialyzed into PBS using Slide-A-Lyzer Cassettes (ThermoFisher 66382), quantified by measuring A_280_ with a Nanodrop instrument (ThermoFisher), and analyzed for purity by separation on 4 to 12% Bis-Tris NuPage Gels (ThermoFisher NP0323BOX) followed by visualization with Gel Code Blue Stain (ThermoFisher 24590). Avi-Tagged RBD proteins were biotinylated using the BirA500 biotin-protein ligase standard reaction kit (Avidity EC 6.3.4.15) according to the manufacturer's instructions, as described previously (44).

### Mouse Experimentation.

Ten-week-old C57BL/6 J male and female mice or K18-hACE2 transgenic mice expressing the human ACE [B6. Cg-Tg (K18-ACE2)2Prlmn/J,034860] were obtained from the Jackson Laboratory (JAX) and housed in a standard BSL1 facility at the Rockefeller Comparative Biosciences Center and regular chow diet, water ad libitum under a 12-h light–dark cycle. The mouse studies were performed in compliance with the animal protocol 21042-H and the Rockefeller University Institutional Animal Care and Use Committee (IACUC). The mice were prebled by the submandibular route and arranged into groups of mixed sex.

### Recombinant Protein Immunization.

Ten-week-old C57BL/6 (The Jackson Laboratory Strain #000664) mice of both sexes, cared for as described above, were immunized using RBD, RBD-RBD fusion dimers, RBD-Fc, or RBD-LzS fusion proteins mixed with Enhanced Magic Mouse Adjuvant using the manufacturer’s protocol (Creative Diagnostics Cat#CDN-A001E). Mice were randomly grouped and antigens were administered by intramuscular injection (a total of 10 µg RBD in 50 µL PBS mixed with 50 µL Enhanced Magic Mouse Adjuvant) with 5 µg injected per hind limb. The mice were injected again with the same antigen/adjuvant combinations 3 wk later. Blood samples were taken at 0, 3, 5, 7, 11, and 12 wk after the first antigen injection.

### AAV Immunization.

Ten-week-old C57BL/6 J or K18-hACE2 were injected intramuscularly with a single dose of a single rAAV (rAAV-RBD_1_-LzS, or rAAV-RBD_Wu_-LzS, 50 μL, 2.5 × 10^10^ vg/mouse, left hind limb). Alternatively, mice received both rAAV-RBD_1_-LzS and rAAV-RBD_Wu_-LzS (1.25 × 10^10^ vg each, combined in a single 50 μL injection (2.5 × 10^10^ vg/ mouse total) left hind limb. In some experiments, rAAV-RBD_1_-LzS and rAAV-RBD_Wu_-LzS (1.25 × 10^10^ vg/mouse) were injected separately in the left and right hind limb. Serum samples were obtained at various time points by submandibular bleeds.

To determine the persistence and coexpression of antigens in individual cells in mixed AAV-based immunogen experiments, 10-wk-old C57BL/6 J or K18-hACE2 were injected intramuscularly with a single dose of a single rAAV encoding mNeonGreen or mScarlet-I at 50 μL 2.5 × 10^10^ vg/mouse. Alternatively, 1.25 × 10^10^ vg of the AAVs encoding mNeonGreen and mScarlet-I were combined in a single 50 μL injection (2.5 × 10^10^ vg/mouse total) and injected in the left hind limb of each mouse. These mice were euthanized at each time point and muscle tissue dissected for embedding, thin-sectioning and microscopy to detect mNeonGreen and mScarlet-I expression.

### Sectioning and Microscopy.

Mice were euthanized with a CO_2_ overdose, and the musculature of the left and right hind limbs was dissected. Muscle tissues were placed in dry ice mixed with 2-methylbutane (isopentane, Fisher Scientific, Cat# 60-048-072), transferred into plastic molds (Fisher Scientific, Tissue-Tek Cryomold, Cat# NC9511236), embedded in optimal cutting temperature (OCT) compound (Fisher Scientific, Tissue-Tek OCT, Cat# NC1029572), cryosectioned at 5 μm, and stained with DAPI (Fisher Scientific, Cat# 62247, Lot SF3605141) nuclear stain at 1:1,000 dilution in PBS for 5 min. Images were acquired at a fixed exposure on a BX60 fluorescence microscope (Olympus America Inc.) equipped with a motorized stage (Prior Scientific Instruments Ltd.) and a CC12 camera (Olympus America Inc.).

### Analysis of Cross-Reactive B Cell Elicitation.

Nine-week-old female C57BL/6 J mice (The Jackson Laboratory, Strain #000664) cared for as described previously were randomly sorted into three groups of 5. Mouse groups were immunized with 3 μg of RBD_1_-RBD_1_, RBD_Wu_-RBD_Wu_, or RBD_1_-RBD_Wu_ fusion dimers. Three control mice were immunized with 12.5 μg of an irrelevant NP-OVAL antigen (Biosearch Technologies N-5051-10). Immunizations were carried out in both footpads, using in a 25-μL volume containing 8.3 μL of 2% Alhydrogel (Invivogen VAC-ALU-250) and 1× PBS. On day 14 postimmunization, the draining popliteal lymph nodes from each mouse were pooled in 200 μL of 1× PBS and mechanically disrupted to create a single-cell suspension and cells recovered by centrifugation at 350 g for 5 min.

Each of the biotinylated RBD_1_ and RBD_Wu_ baits (5 μg/mL) were incubated with two fluorophore-conjugated streptavidins (1 μg/mL) in separate reactions for 30 min. RBD_1_ was conjugated to streptavidin-BV711 (BD Biosciences 563262) and streptavidin-BUV661 (BD Biosciences 612979). RBD_Wu_ was conjugated to streptavidin-APC (BioLegend 405207) and streptavidin-PE (BD Biosciences 554061). These four RBD-streptavidin conjugates were pooled, and a 1:200 dilution of Mouse BD Fc Block (BD Biosciences 553142) and 1:500 dilution of Zombie NIR (BioLegend 423106) were added to the mixture. Popliteal lymph node cells were then stained with these RBD-streptavidin complexes for 30 min and centrifuged at 350 g for 5 min. Next, cells were resuspended in 200 μL of a mixture containing each of the following antibodies at a 1:200 dilution for 20 min: 1) anti-CD4-APC-eFluor780 (ThermoFisher 47004282), 2) anti-CD8a-APC-eFluor780 (ThermoFisher 47008182), 3) anti-Ly-6G/Ly-6C-APC-eFluor780 (ThermoFisher 47593182), 4) anti-NK1.1-APC-eFluor780 (ThermoFisher 47594182), 5) anti-F4/80-APC-eFluor780 (ThermoFisher 47480182), 6) anti-CD38-AlexaFluor 700 (ThermoFisher 56038182), 7) anti-CD95-BUV563 (BD Biosciences 741292), and 8) anti-CD45R/B220-BUV805 (BD Biosciences 748867). Cells were washed with 200 μL of 1× PBS, filtered through a 40 μM strainer (Fisher Scientific 08-771-1) and fluorescence profiles acquired on a BD FACSymphony system (BD Biosciences). GC B cells were defined as B220^+^, CD4^−^, CD8^−^, Ly-6G/Ly-6C^−^, NK1.1^−^, F4/80^−^, CD38^−^, CD95^+^ and the RBD_1_ and RBD_Wu_ binding cells in this fraction were enumerated.

### SARS-CoV-2 Challenge Experiments.

Mice (K18-hACE2) were weighed and anesthetized via intraperitoneal injection of a mixture of ketamine (80 mg/kg) and xylazine (8.8 mg/kg). Then, 30 µL of viral inoculum (20,000 PFU of SARS-CoV-2 WA.1 or SARS-CoV-2 BA.5) was applied to one nostril. At day 3 postinfection, the mice were weighed, humanely euthanized and lungs dissected and weighed. The left lung lobe was fixed overnight in 10% formalin (Sigma-Aldrich, Cat# HT5011280-a4L) and preserved in 75% ethanol (Koptec). The right lung was homogenized in Trizol LS reagent (Ambion, Life Technologies, Cat# 10296028) and total RNA extracted by phase separation using chloroform (Sigma-Aldrich, Cat# C2432-500ML). RNA in the aqueous phase was precipitated using isopropanol (Sigma-Aldrich, I9516-500ML) and pelleted RNA washed with ice-cold 75% ethanol. The RNA was dissolved in nuclease-free water, and the number of viral genomes per microgram of the right lung was measured by the qRT-PCR using 1-Step Kit, PowerSYBR Green RNA-to-CT (ThermoFisher Scientific), and StepOne Plus Real-Time PCR system (Applied Biosystems). The primers used were 2019-nCoV_N1-F: 5′-GACCCCAAAATCAGCGAAAT-3′ and 2019-nCoV_ N1-R: 5′-TCTGGTTACTGCCAGTTGAATCTG-3′, targeting RNA sequences that encode the nucleocapsid protein of SARS-COV-2 WA.1 and BA.5. A standard, 2019-nCoV_N_Positive Control 10006625 was obtained from IDT.

### SARS-CoV-2 Production and Titration.

SARS-CoV-2 strains USA-WA1/2020 and BA.5 were obtained from BEI Resources (catalog no. NR-52281 and NR-58616, respectively). The USA-WA1/2020 variant was amplified in Caco-2 cells, by infection at a multiplicity of infection (MOI) of 0.05 plaque forming units (PFU)/cell and incubation for 5 d at 37 °C. The BA.5 variant was amplified in VeroE6 cells obtained from the ATCC that were engineered to stably express TMPRSS2 (VeroE6_TMPRSS2_). These cells were infected with BA.5 at a MOI = 0.05 PFU/cell and incubation for 4 d at 33 °C. Virus-containing supernatants were harvested, clarified by centrifugation (3,000*g* × 10 min), filtered using a disposable vacuum filter system with a 0.22-μm membrane (Corning, Cat# 430320), and stored at −80 °C. Virus titers were measured by standard plaque assay (PA) on VeroE6 cells obtained from Ralph Baric (VeroE6_UNC_). Briefly, 500 µL of serial 10-fold virus dilutions in Opti-MEM (Gibco Cat# 31985070) were used to infect 4 × 10^5^ cells seeded the day prior into wells of a 6-well plate. After 1.5 h adsorption, the virus inoculum was removed, and cells were overlayed with DMEM containing 10% FBS with 1.2% microcrystalline cellulose (Avicel, FMC BioPolymer, Cat RC-581). Cells were incubated for 5 d at 37 °C, followed by fixation with 7% formaldehyde (Fisher Chemical, Cat F79-1) and crystal violet (Fisher Chemical, Cat# C581-100) staining for plaque enumeration. All SARS-CoV-2 experiments were performed in a biosafety level 3 laboratory. Virus stock titers on VeroE6_UNC_ cells for SARS-CoV-2 USA-WA1/2020 (P3) were 7.8 × 10^6^ PFU/mL and for SARS-CoV-2 BA.5 (P2) were 2.9 × 10^6^ PFU/mL.

### SARS-CoV and SARS-CoV-2 Pseudotyped HIV-1 Viruses.

SARS-CoV and SARS-CoV-2 spike pseudotyped HIV-1/NanoLuc particles were produced as described previously (41). Briefly, 12 × 10^6^ 293 T cells were cotransfected with 12.5 μg pHIV-_1NL4-3_ΔEnv-NanoLuc reporter and 4.5 μg plasmids expressing the spike proteins of SARS-CoV, SARS-CoV-2 (Wuhan-hu-1), or BA.5 (SinoBiological) using PEI at a ratio of 4:1 PEI:DNA in Opti-MEM™ (Gibco 31985070). At 48 h posttransfection, supernatants from the cells were harvested, clarified by centrifugation, and filtered (0.22 μm, Millipore S2GPU05RE). Aliquots were stored at −80 °C, and titers were determined by serial dilution and infection of HT1080/ACE2.cl14 cells, with measurement of NanoLuc luciferase activity 48 h postinfection using the Nano-Glo Luciferase Assay System (Promega N1150) and a Glomax Navigator luminometer (Promega).

### Neutralization Assays.

Mouse serum was tested for neutralizing potency against SARS-CoV, SARS-CoV-2, and its variants using the spike-pseudotyped HIV-1-based assay described (41) previously. Briefly, following heat inactivation at 55 °C for 30 min, mouse serum samples were fourfold or fivefold serial diluted using a Pipetmax (Gilson GFAM0072) and incubated with spike-pseudotyped HIV-1 reporter virus for 1 h at 37 °C. The serum-pseudotype virus mixture was then added to 1 × 10^4^ HT1080/ACE2.cl14 cells seeded in 96-well black plates (Costar 3916). At 48 h after infection, the cells were washed with PBS and lysed in Luciferase Cell Culture Lysis reagent (Promega E1531). Luciferase activity was measured using the Nano-Glo Luciferase Assay System (Promega N1150) and a Glomax Navigator luminometer (Promega) or the ClariostarPlus Microplate Reader (BMG LabTech). The relative luminescence units (RLUs) were plotted as a decimal fraction of those obtained in wells on the same plate that were infected in the absence of mouse serum. The normalized data were plotted in GraphPad Prism, and the NT_50_ was calculated using four-parameter nonlinear regression.

### Neutralization Depletion Assays.

Aliquots (4 µL) of mouse serum were incubated with 10 µg RBD-6xHis in 30 µL PBS at 4 °C with shaking. After 30 min, 10 µL His-Tag Dynabeads (ThermoFisher 10103D) were added, and the mixture was incubated for an additional 30 min at 4 °C with shaking. After magnetic separation of the beads, the supernatant was transferred to a fresh 96-well plate and serially diluted for assessment of neutralization potency against pseudotyped virus using the assay described above.

### Antibody: RBD-NanoLuc Binding and Competition Assays.

Fourfold serial dilutions of mouse serum were incubated with 10 ng of an RBD-NanoLuc fusion protein at 4 °C with shaking. After 30 min, 10 µL Protein G Dynabeads (ThermoFisher 10004D) were added, and the samples were incubated for 15 min at 4 °C with shaking. The Dynabeads were magnetically separated, washed three times with 100 µL PBS, and then incubated in 50 µL Luciferase Cell Culture Lysis reagent (Promega E1531). Bead-bound NanoLuc luciferase activity was measured using the Nano-Glo Luciferase Assay System (Promega N1150) and a Glomax Navigator luminometer (Promega).

Serum concentrations yielding RBD-NanoLuc binding capacities of 1 × 10^5^ to 1 × 10^6^ RLU were used for competition assays. For the competition assays, mouse serum was incubated with a fourfold serially diluted unlabelled RBD (0 to 500 ng) and 10 ng of an RBD-NanoLuc fusion protein for 30 min at 4 °C with shaking; then, 10 µL Protein G Dynabeads were added, and the samples were incubated for an additional 15 min at 4 °C with shaking. The Dynabeads were separated using a magnet, washed and then bead-bound NanoLuc luciferase activity measured as described above. Bead-bound NanoLuc luciferase activities were plotted as decimal fraction of those obtained in the absence of unlabelled RBD competitor. The normalized data were plotted in GraphPad Prism, and the IC_50_ was calculated using four-parameter nonlinear regression.

### Statistical Analysis.

Statistical analysis was performed using Prism 9.0 (GraphPad Software). Mean values were compared between groups by Student’s *t* test and two-tailed Mann–Whitney *U* tests.

## Supplementary Material

Appendix 01 (PDF)

## Data Availability

Numerical data have been deposited on Figshare (DOI: 10.6084/m9.figshare.24585138) (45). All other study data are included in the article and/or *SI Appendix*.
