# Supplementary material for: Heteromultimeric sarbecovirus receptor binding domain immunogens primarily generate variant-specific neutralizing antibodies
Source: Proc Natl Acad Sci U S A. 2023 Dec 14;120(51):e2317367120. doi: 10.1073/pnas.2317367120 (PMC10740387; doi:10.1073/pnas.2317367120)
Supplement: Supplementary file 1 — Appendix 01 (PDF) [file pnas.2317367120.sapp.pdf]

PNAS

Supporting Information for

Heteromultimeric sarbecovirus receptor binding domain immunogens primarily generate variant-specific neutralizing antibodies.

Trinity Zang, Edmund Osei Kuffour, Viren A. Baharani, Marie Canis, Fabian Schmidt, Justin Da Silva, Alexander Lercher, Pooja Chaudhary, Hans-Heinrich Hoffmann, Anna Gazumyan, Ileana C. Miranda, Margaret R. MacDonald, Charles M. Rice, Michel C. Nussenzweig, Theodora Hatziioannou and Paul D. Bieniasz

Corresponding authors: Paul D. Bieniasz and Theodora Hatziioannou
Email: pbieniasz@rockefeller.edu, thatziio@rockefeller.edu

This PDF file includes:

Figures S1 to S10

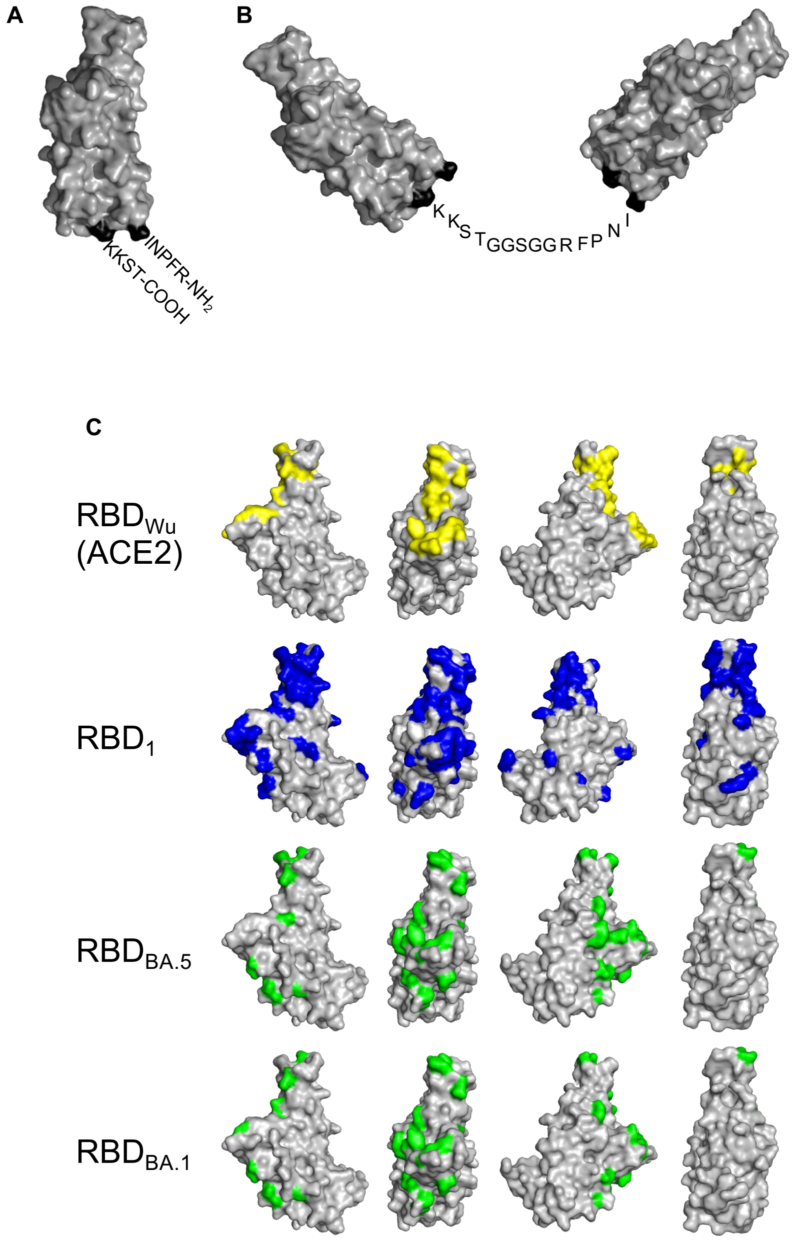

Fig. S1. Configuration of genetically fused dimeric RBD immunogens. (A) The NH2 and COOH termini of the conformationally discrete SARS-CoV-2 RBD are indicated in black (B) representation of a generic, genetically fused SARS-CoV-2 RBD dimer incorporating amino acids 328-531 of each RBD joined by a GGGSGG linker (C) Four views differing by 90° rotation of the SARS-CoV-2$_{Wu}$ RBD with amino acids that constitute the ACE2 binding site (yellow) or differ in RBD$_1$ (blue), RBD$_{BA.5}$ (green), and RBD$_{BA.1}$ (red).

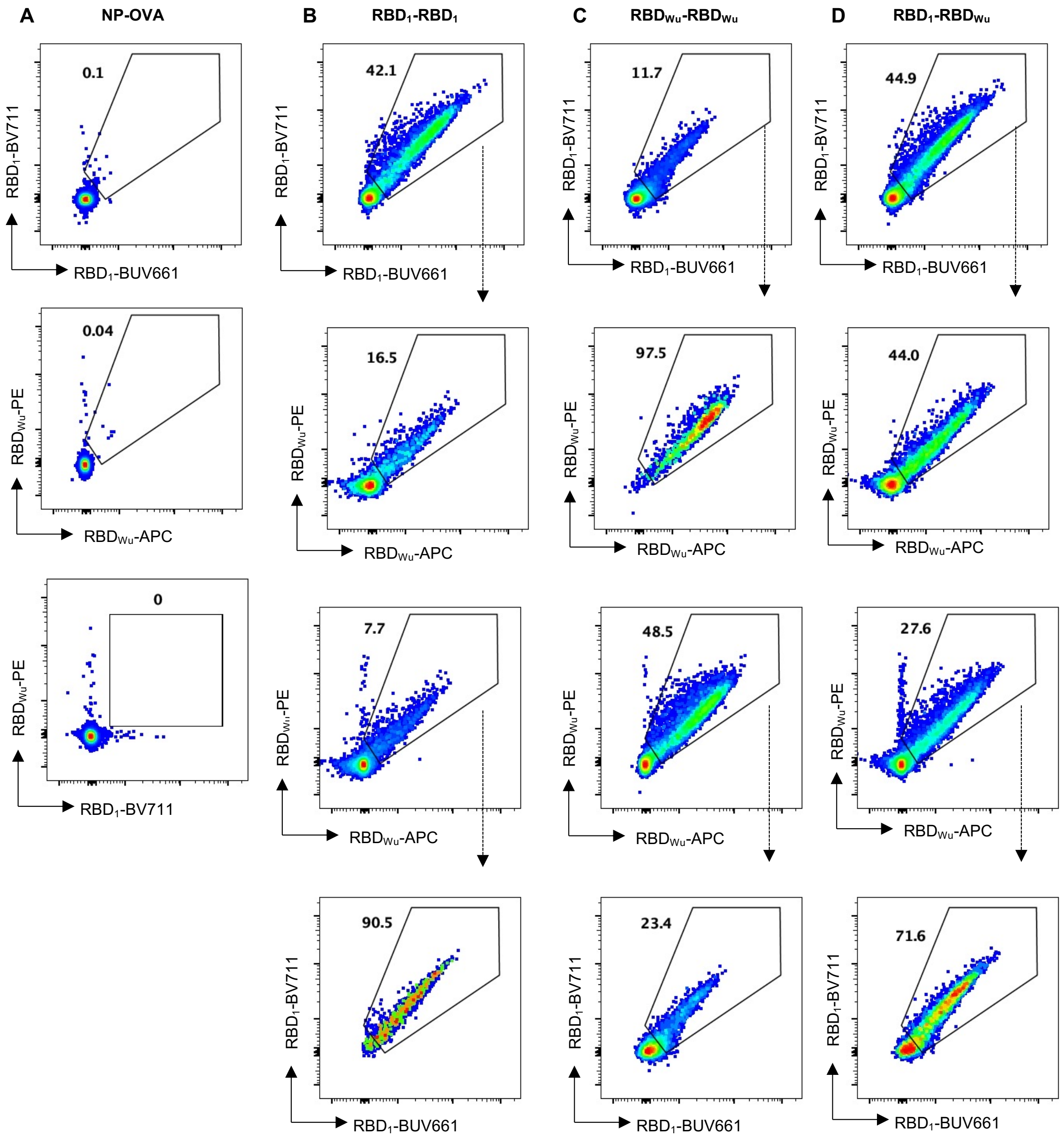

Fig. S2. Extent of cross reactivity in B cells responding to RBD homodimers and heterodimers. (A-D) Examples of FACS analysis of mouse popliteal lymph node GC B-cells (B220$^+$, CD4$^-$, CD8$^-$, Ly-6G/Ly-6C$^-$, NK1.1$^-$, F4/80$^-$, CD38$^-$, CD95$^+$) binding to fluorophore conjugated RBD$_1$ and RBD$_{Wu}$. Mice were footpad immunized with NP-OVA as a control (A), or RBD$_1$-RBD$_1$ (B), RBD$_{Wu}$-RBD$_{Wu}$ (C), or RBD$_1$-RBD$_{Wu}$ (D). For (A), the upper panel shows overall GC B cells binding to RBD$_1$, labelled with two different streptavidin fluorophores, middle panel shows binding to RBD$_{Wu}$, labelled with two different streptavidin fluorophores, lower panel shows overall B cell binding to RBD$_1$ and RBD$_{Wu}$ each labelled with a single streptavidin fluorophore. For (B-C): Upper row shows overall GC B cell binding to RBD$_1$, labelled with two different streptavidin fluorophores, second row shows binding to RBD$_{Wu}$ labelled with two different streptavidin fluorophores after gating on RBD$_1$ binding cells. Third row shows overall B cell binding to RBD$_{Wu}$ labelled with two different streptavidin fluorophores. Fourth row shows GC B cell binding to RBD$_1$ labelled with two different streptavidin fluorophores, after gating on RBD$_{Wu}$ binding cells.

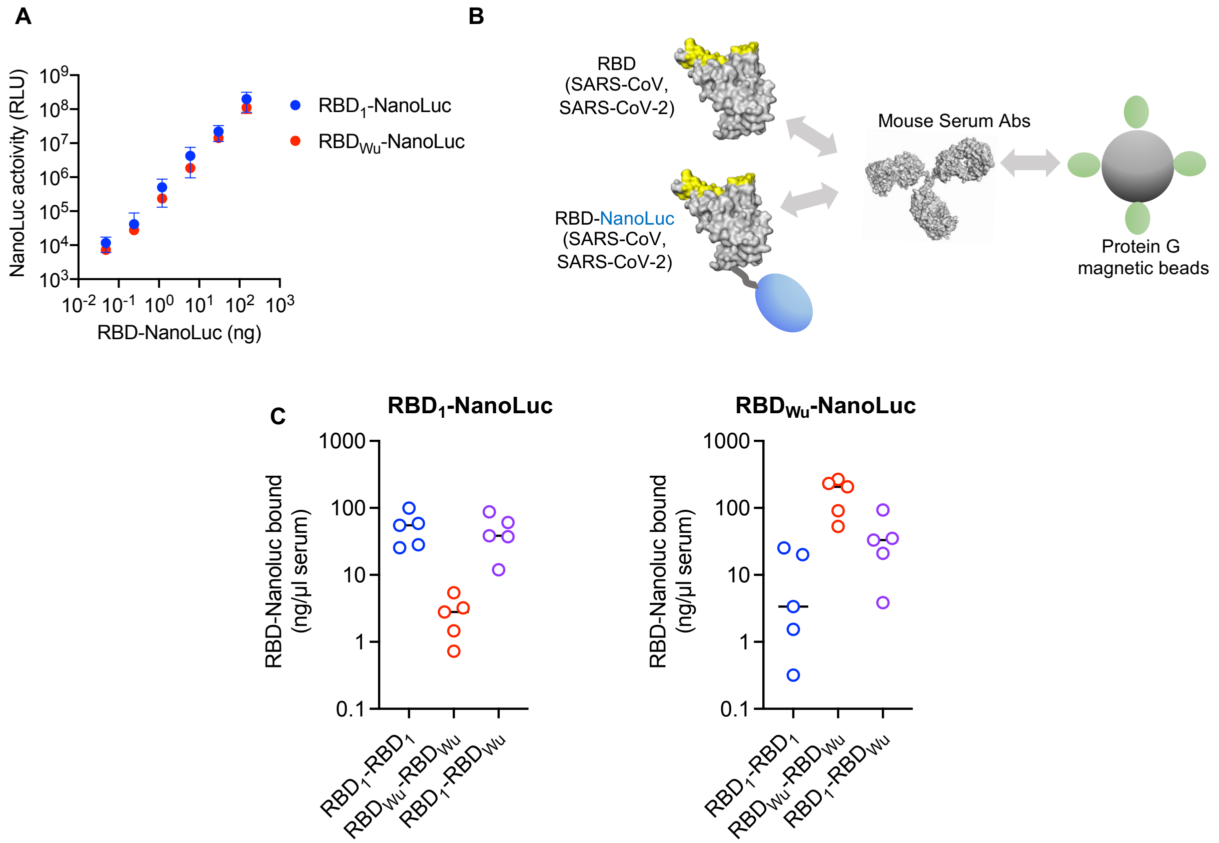

Fig. S3. Competitive RBD-binding antibody assay (A) RBD-NanoLuc fusion proteins expressed in Expi293 cells were purified and NanoLuc activity is plotted as a function of protein amount (ng) (B) Representation of assay procedure, mouse sera are incubated with varying amounts of unlabelled RBD, then a saturating amount (10ng) of RBD-NanLuc is added, and antibody/RBD/RBD-NanoLuc complexes separated with protein-G Dynabeads before measurement of bead-bound NanoLuc activity. (C) RBD-NanoLuc and RBD_{Wu}-NanoLuc binding capacity of mouse sera following immunization with the RBD dimers indicated on the x axis.

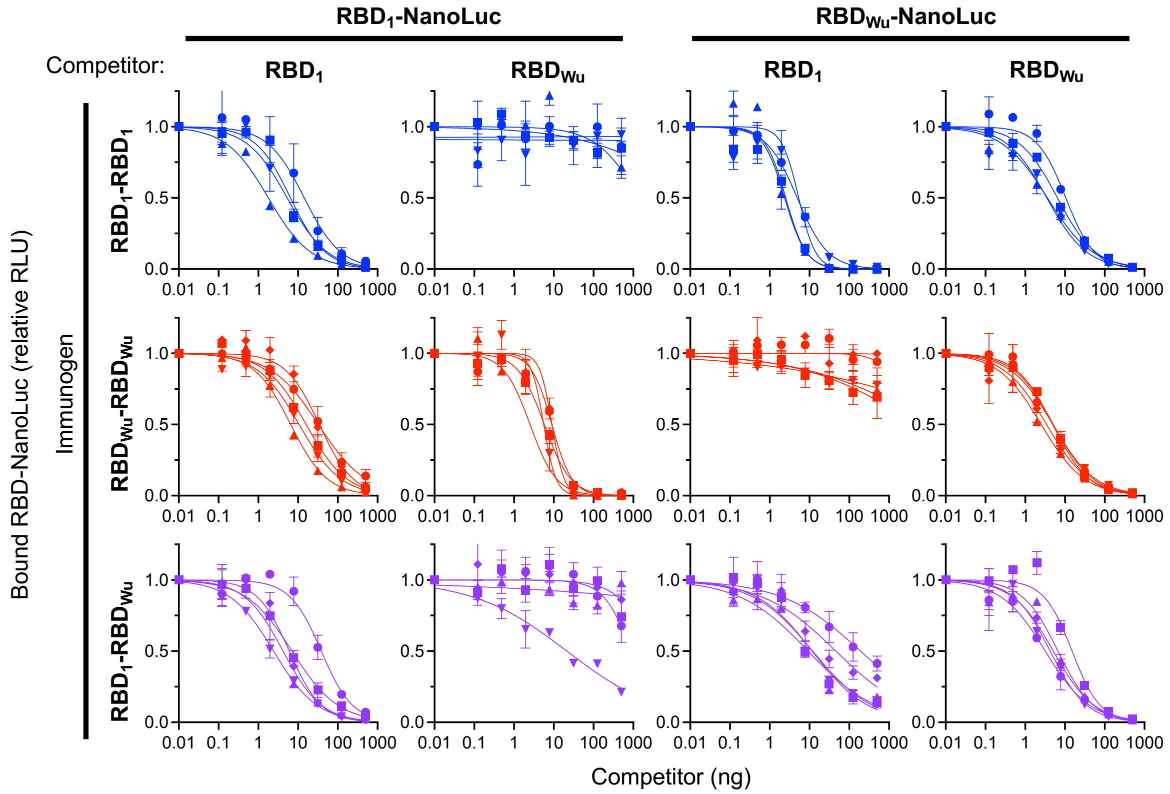

Fig. S4. Measurement of RBD_1/RBD_{Wu} cross-reactive serum antibodies. Inhibition curves for RBD_1 and RBD_{Wu} competition with RBD_1-NanoLuc and RBD_{Wu}-NanoLuc for binding to serum antibodies from mice immunized with RBD_1-RBD_1 (upper row), RBD_{Wu}-RBD_{Wu} (middle row) or RBD_1-RBD_{Wu} (bottom row). Antibody-RBD/RBD-NanoLuc complexes formed in solution were captured with protein-G Dynabeads and bound NanoLuc activity measured. The decimal fraction of RBD-NanoLuc activity bound to protein G dynabeads in the absence of RBD competitor is plotted. Five mice were evaluated for each group and error bars indicate the range of two technical replicates.

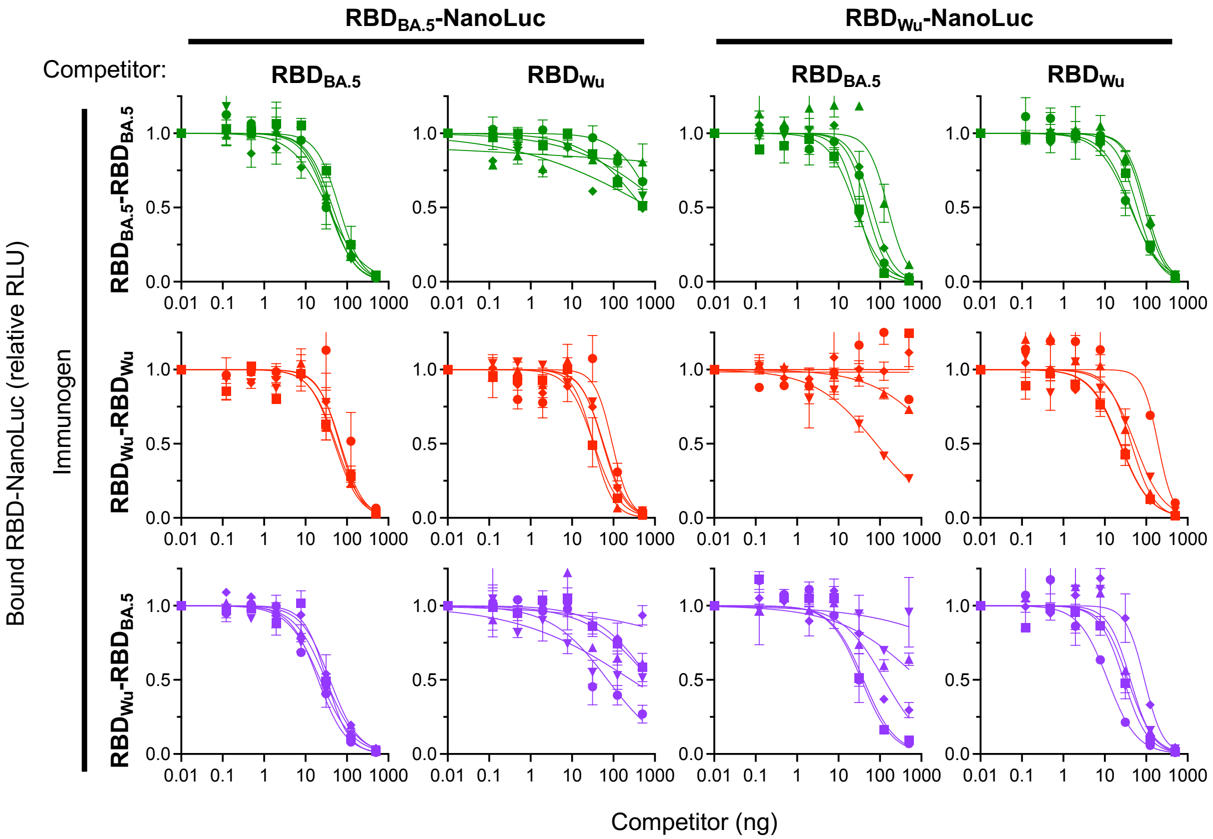

Fig. S5. Measurement of RBD$_{BA.5}$/RBD$_{Wu}$ cross-reactive serum antibodies. Inhibition curves for RBD$_{BA.5}$ and RBD$_{Wu}$ competition with RBD$_{BA.5}$-NanoLuc and RBD$_{Wu}$-NanoLuc for binding to serum antibodies from mice immunized with RBD$_{BA.5}$-RBD$_{BA.5}$ (upper row), RBD$_{Wu}$-RBD$_{Wu}$ (middle row) or RBD$_{Wu}$-RBD$_{BA.5}$ (bottom row). Antibody-RBD/RBD-NanoLuc complexes formed in solution were captured with protein-G Dynabeads and bound NanoLuc activity measured. The decimal fraction of RBD-NanoLuc activity bound to protein G dynabeads in the absence of RBD competitor is plotted. Five mice were evaluated for each group and error bars indicate the range of two technical replicates.

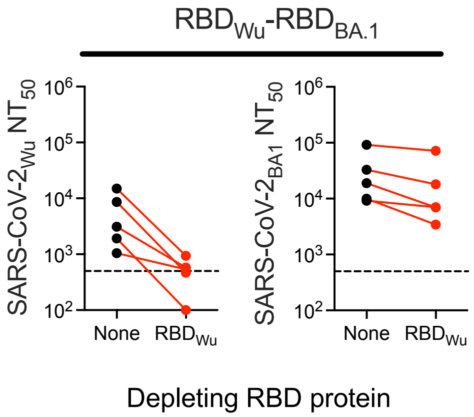

Fig. S6. $RBD_{BA.1}$ and RBD_{Wu} cross-reactive serum antibodies in heterodimer immunized mice. Neutralizing antibody titers (NT_{50}) against SARS-CoV-2_{Wu} and SARS-CoV-$2_{BA.1}$ in sera from mice immunized with RBD_{Wu}-$RBD_{BA.1}$ following mock depletion (None) or depletion with the RBD_{Wu} protein.

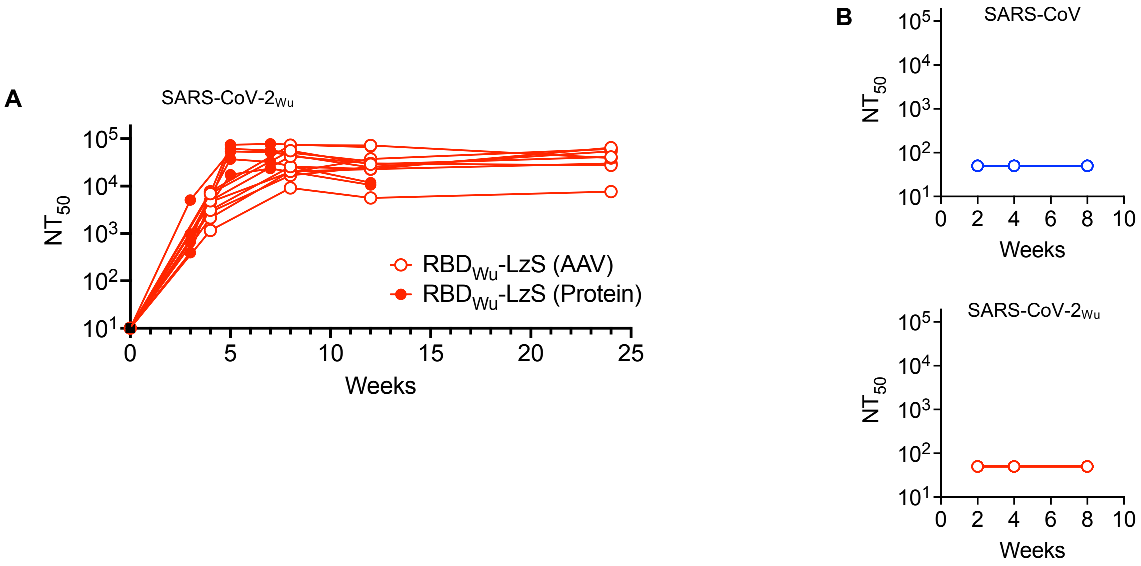

Fig. S7. Neutralizing antibodies to SARS-CoV-2_{Wu} after a single dose rAAV RBD-LzS immunization. (A) Neutralizing titers (NT_{50}) against SARS-CoV-2_{Wu} pseudotype in the 24 weeks following immunization with a single dose of rAAV RBD_{Wu}-LzS (A) or in the 12 weeks following initiation of immunization with RBD_{Wu}-LzS protein (two doses at week 0 and week 3). (B) Neutralizing titers (NT_{50}) against SARS-CoV and SARS-CoV-2_{Wu} pseudotype in the 8 weeks following control immunization with a single dose of rAAV mNeonGreen.

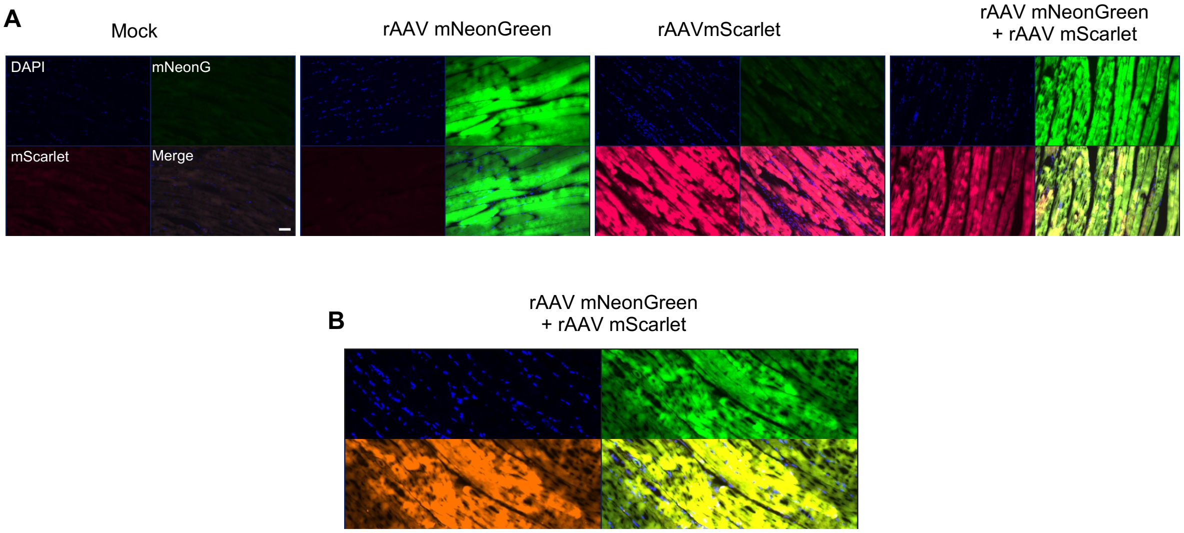

Fig. S8. Long-term co-expression of reporter genes in individual muscle fibers after co-injection with two rAAV vectors. (A) Expression of mNeonGreen and mScarlet proteins in hind limb muscles dissected from mice injected with rAAV mNeonGreen, rAAV mScarlet or both as indicated, at 8 weeks after injection. (B) Expression of mNeonGreen and mScarlet proteins in a hind limb muscle at 24 weeks after co-injection with both rAAV mNeonGreen and rAAV mScarlet.

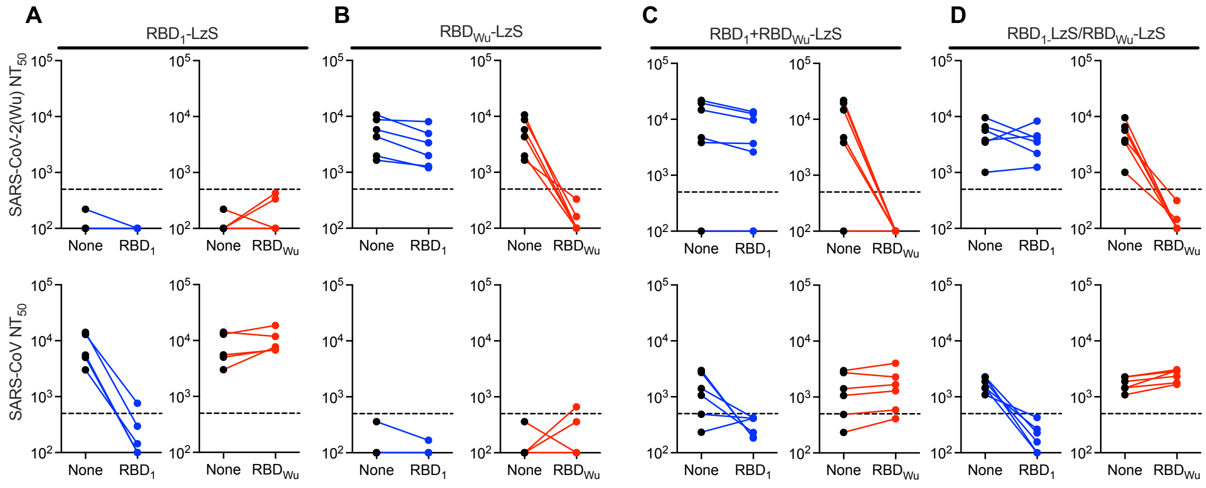

Fig. S9. RBD_1 and RBD_{Wu} cross-reactive neutralizing serum antibodies in rAAV RBD_1-LzS and rAAV RBD_{Wu}-LzS immunized mice. (A-D) Neutralizing antibody titers (NT_{50}) against SARS-CoV-2_{Wu} (upper panels) and SARS-CoV (lower panels) in sera from mice immunized with rAAV RBD_1-LzS ($2.5x10^{10}$ viral genomes (vg)) alone (A), rAAV RBD_{Wu}-LzS ($2.5x10^{10}$ vg) alone (B) a mixture of rAAV RBD_1-LzS and rAAV RBD_{Wu}-LzS ($1.25x10^{10}$ vg each in a single injection) (C) or rAAV RBD_1-LzS and rAAV RBD_{Wu}-LzS ($1.25x10^{10}$ vg separately in each hindlimb) (D). NT_{50} was measured following mock depletion (None) or depletion with the indicated RBD protein.

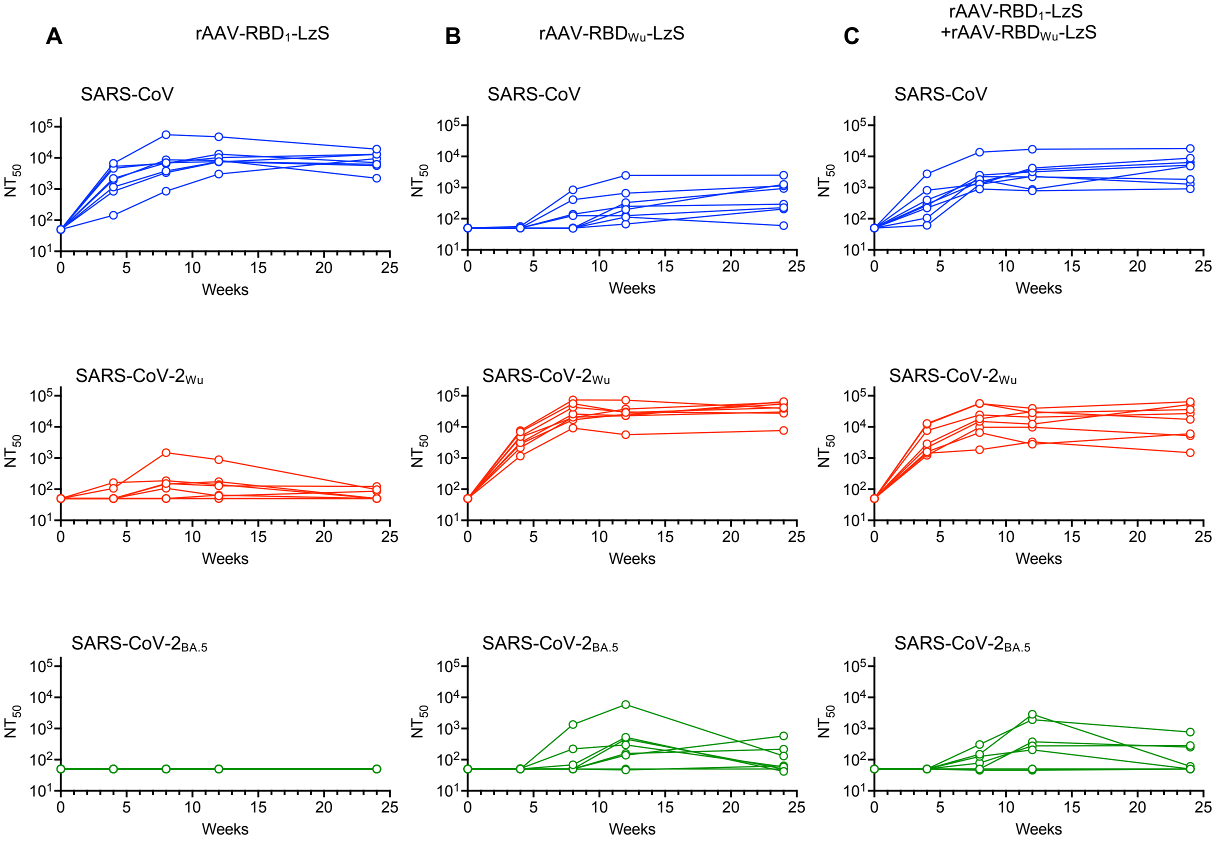

Fig. S10. Neutralizing antibodies following a single dose rAAV RBD-LzS immunization. (A-C) Neutralizing titers (NT_{50}) against SARS-CoV, SARS-CoV-2_{Wu} and SARS-CoV-$2_{BA.5}$ pseudotypes in mouse sera in the 24 weeks following immunization with a single dose of rAAV RBD_1-LzS ($2.5x10^{10}$ vg) (A), rAAV RBD_{Wu}-LzS ($2.5x10^{10}$ vg) (B), or a mixture of both ($1.25x10^{10}$ vg each in a single hindlimb injection) (C).
